# Bamlanivimab Reduces ED Returns and Hospitalizations and May Reduce COVID-19 Burden on Low-resource Border Hospitals

**DOI:** 10.5811/westjem.2021.10.52668

**Published:** 2022-03-17

**Authors:** Faith C. Quenzer, Andrew T. Lafree, Londyn Grey, Sukhdeep Singh, Cameron Smyers, Bruce Balog, Henry Montilla Guedez, Kaitlin McIntyre, Sharon Wulfovich, Juli Ramirez, Talia Saikhon, Christian Tomaszewski

**Affiliations:** * San Diego State University School of Public Health, San Diego, California; † University of California – San Diego, School of Medicine, Department of Emergency Medicine, San Diego, California; ‡ El Centro Regional Medical Center, Department of Emergency Medicine, El Centro, California; § Louisiana State University Health Sciences Center, School of Medicine, Shreveport, Louisiana

## Abstract

**Introduction:**

To evaluate the effectiveness of bamlanivimab at reducing return emergency department (ED) visits in primarily Latinx/Hispanic patients with mild or moderate coronavirus disease 2019 (COVID-19). Secondary aims were to evaluate the prevention of subsequent hospitalizations and deaths in a resource-limited United States (U.S.)-Mexico border hospital.

**Methods:**

We conducted a retrospective, open-label interventional study on 270 eligible adult patients diagnosed with mild-moderate severe acute respiratory syndrome coronavirus 2 (SARS-CoV-2) infection who met criteria for receiving bamlanivimab from November 1, 2020 to January 31, 2021. The main outcomes of 14-day return visits to the ED and hospitalizations due to COVID-19 were compared between two groups – those who received bamlanivimab (exposed group) and those who did not receive bamlanivimab (unexposed group). Outcomes were analyzed through chi-square tests followed by multivariate regression modeling to adjust for patient demographics, characteristics, and comorbidities.

**Results:**

There were 136 COVID-19 patients who received bamlanivimab in the ED prior to discharge and an unexposed group of 134 COVID-19 patients who were evaluated and discharged from the ED without receiving bamlanivimab. Overall, mean age was 61.7 (S.D. +/−13.9) years, mean body mass index (BMI) 31.0 (S.D. +/−6.6) kg/m^2^, 91.5% identified as Latinx/Hispanic, 51.9% male, and 80.7% reported at least one comorbidity. Most commonly reported comorbidities were obesity (22.6%), hypertension (59.6%), and diabetes (41.1%). The bamlanivimab group had a 22.8% (mean estimate = 0.7717, 95% CI [0.6482, 0.8611]) risk reduction or 84.4% (0.3030, 95% CI = 0.166, 0.554, p=.0001) absolute reduction of ED return visits within 14 days compared to controls after adjusting for chronic kidney disease. The bamlanivimab group had 19.0% (mean estimate=0.8097, 95% CI [0.6451, 0.9087]) risk reduction or 96.2% (0.235, 95% CI 0.100, 0.550, p=0.0008) absolute reduction of subsequent hospitalizations compared to unexposed patients after adjusting for diabetes status.

**Conclusion:**

Bamlanivimab infusions for high-risk COVID-19 patients in the ED substantially reduced the risk of return visits to the ED and hospitalizations in our primarily Latinx/Hispanic population. Monoclonal antibody infusions may help reduce hospital utilization during COVID-19 surges at U.S.-Mexico border hospitals.

## INTRODUCTION

Severe acute respiratory syndrome coronavirus 2 (SARS-CoV-2), which is responsible for the current coronavirus disease 2019 (COVID-19) pandemic, has burdened healthcare systems across the United States to their breaking point because of rapid influxes of critically ill patients who require weeks of hospitalization, intensive care resources, and healthcare personnel.^1^ Hospitals have been forced to admit patients beyond maximum capacity and have stretched healthcare personnel responsibilities beyond what are normally considered safe levels.^1^ Hospitals near the US-Mexico border are particularly vulnerable to these problems because of inadequate healthcare infrastructure, healthcare resources, and healthcare workers at baseline.^2^ As a result, COVID-19 has disproportionately impacted low-resourced and rural hospitals compared to their urban counterparts, and COVID-19 mortality is as much as three times higher in hospitals with fewer intensive care unit (ICU) beds available.^1^,^3^

Resource challenges are further compounded by cross-border traffic that makes populations at the US-Mexico border more vulnerable to surges of communicable diseases such as COVID-19.^2^,^4^ Additionally, border populations, which are predominantly Latinx/Hispanic, are more likely to have underlying chronic conditions such as diabetes, hypertension, coronary artery disease, chronic kidney disease, and chronic lung disease.^2^,^5^ These comorbidities make these populations more susceptible to severe COVID-19 complications such as hospitalization, invasive ventilation, and death.^6^–^7^ Compared to White non-Hispanics, Latinx/Hispanics account for four times the hospitalizations and nearly three times the deaths due to COVID-19 in the US.^2^,^6^–^7^

Our study was performed at the El Centro Regional Medical Center (ECRMC), which is located 12 miles north of the US-Mexico border in Imperial County, California, and serves a predominantly Latinx/Hispanic patient population. Imperial County has the second-highest number of COVID-19 cases and the highest COVID-19 death rate per population (374 people per 100,000) in the entire state of California by the summer of 2020.^8^ Over the course of the year the ECRMC emergency department (ED) saw 3,876 COVID-19 patients, with 1,342 hospital admissions and 336 deaths (ECRMC internal hospital data, 2020). According to the internal, unpublished ECRMC data, the peak of the 2020 COVID-19 pandemic demonstrated ECRMC’s patient load rose to nearly two times the normal patient census and 10 times the normal ICU census. In December 2020 alone, over 40 patients were on ventilators, most in routine medical-surgical rooms. From March–July 2020, during the first COVID-19 surge in Imperial County, 18.7% of the 497 COVID-19 patients who were admitted to ECRMC expired; during the second surge from November 2020–January 2021 mortality of admitted COVID-19 patients increased to 37.0% (ECRMC, internal hospital data, 2020). This increased mortality rate may partly reflect the resources available to the local hospital system relative to the burden of COVID-19 faced during the significantly worse second surge and the indirect effects of hospital saturation on patient outcomes.

**Population Health Research Capsule**

What do we already know about this issue?

*Latinx patients have the worst COVID-19 outcomes. Bamlanivimab, an outpatient monoclonal antibody treatment, can help prevent COVID hospitalizations and improve outcomes.*

What was the research question?

*Could bamlanivimab prevent ED return visits and hospitalizations in high-risk Latinx COVID-19 patients at a border hospital?*

What was the major finding of the study?

*Bamlanivimab given to high-risk, Latinx COVID-19 patients at a border hospital ED decreased ED return visits and hospitalizations.*

How does this improve population health?

*Monoclonal antibodies administered in a low-resource ED may help decrease ED return visits in high-risk Latinx patients and hospitalizations during a surge.*

The response to the COVID-19 pandemic in limited-resource border hospitals such as ECRMC has highlighted major challenges. With the possibility of new surges from more variants there is still a need for innovative, rapidly operationalized solutions. In conjunction with the state and local public health authorities, ECRMC has been using monoclonal antibody treatment for high-risk, non-hospitalized patients with mild-to-moderate COVID-19 since November 2020. The primary goal was to reduce subsequent hospitalizations in high-risk patients and alleviate further pressure on a resource-scarce healthcare system. Its use has been predicated on the Emergency Use Authorization (EUA) from the US Food and Drug Administration (FDA).^9^

There have been several strategies using passive immunity to enable the humoral immune response against SARS-CoV-2, some of which include convalescent plasma, immune globulin, and monoclonal antibodies.^10^ Monoclonal antibodies are very specific and highly concentrated antibodies that are laboratory developed to bind and neutralize viruses such as Ebola and rabies.^11^,^12^ For SARS-CoV-2, the first two monoclonal antibody treatments initially available were bamlanivimab and the combination of casirivimab and imdevimab (Regeneron [Regeneron Pharmaceuticals, Inc., Eastview/Tarrytown, NY).^9^,^11^ These monoclonal antibodies are specifically made to attach and neutralize the SARS-CoV-2 surface spike glycoprotein, which binds to the angiotensin-converting enzyme 2 receptors to gain access to cells.^11^,^13^

There is limited evidence from ongoing randomized control trials that these monoclonal antibodies may decrease viral load and the progression of COVID-19 disease in high-risk, non-hospitalized patients with mild or moderate symptoms.^11^,^14^ However, when given to hospitalized patients with severe COVID-19, no significant difference in complications and disease progression has been shown.^11^ On November 9, 2020, the FDA gave these monoclonal antibodies EUA.^9^ Some preliminary trial data suggests that the use of monoclonal antibodies in outpatient treatment of COVID-19 may prevent hospitalization, invasive ventilation/intubation, and death.^13^ More research evaluating how these monoclonal antibodies could impact ED and hospital utilization is needed, especially in the setting of rural or border hospitals with limited resources and a high-risk of increased COVID-19 burden.

The purpose of our study was to evaluate the effectiveness of bamlanivimab in preventing return ED visits, hospitalizations, and mortality within the Latinx/Hispanic population in a border community hospital. Emergency departments can rapidly and easily operationalize systems for early distribution of monoclonal antibodies to SARS-CoV-2 patients who are at high risk for developing severe COVID-19 disease early in the course of their illness. Aside from the costs associated with monoclonal antibodies themselves, infusing monoclonal antibodies early in COVID-19 disease is not personnel or resource intensive. If even modest reductions in subsequent return ED visits and hospitalizations could be demonstrated, targeted, large-scale monoclonal antibody infusions may significantly reduce the burden on these EDs and healthcare systems.

## METHODS

### Study Design

We performed a retrospective, cohort study of the monoclonal antibody bamlanivimab (Eli Lilly and Company, Indianapolis, IN) in non-hospitalized, adult ED patients diagnosed with COVID-19 at a border hospital. The primary outcomes of interest were return visit to the ED within 14 days and subsequent hospitalization in patients who did not receive bamlanivimab (unexposed) vs patients who received bamlanivimab (exposed). Mortality outcomes were also described. We selected the 14-day outcome based on observational reports demonstrating that on average, patients were hospitalized for dyspnea 7–10 days after initial symptoms.^15^ Therefore, after 14 days from initial diagnosis, the likelihood of deterioration should be lessened substantially and the majority of patients ultimately requiring hospitalization would already have been hospitalized. For patients who returned to the ED and were hospitalized we reviewed the entire clinical course.

Per Bledsoe and Worster, we accessed the electronic health records (EHR) database to identify ED visits and hospitalizations. Abstractors were trained in obtaining the necessary data from these various EHR, and cases were selected by criteria that had defined inclusion and exclusion criteria. Quality control of the data was done throughout the data collection by three of the investigators from this study.^16^ This study was institutional review board (IRB #200558) exempt. Patients who were interested in treatment were informed of the risks and benefits of receiving bamlanivimab as outlined in the FDA EUA, and consent for treatment was obtained.

### Inclusion

Included within this study were adult patients (≥18 years) with a laboratory-confirmed diagnosis of COVID-19 detected in our ED or patients who had tested positive at an outside healthcare facility and presented to our ED requesting treatment with monoclonal antibodies. All patients from outside facilities were required to have documentation of a newly positive COVID-19 test within seven days. Screening for inclusion criteria were required prior to treatment for all patients. Eligible ED patients who were diagnosed with COVID-19 in the ED were offered treatment immediately if monoclonal antibodies were available. Patients who had been discharged prior to receiving COVID-19 test results were called back the following day with results. If monoclonal antibodies were available at that time and the patient met eligibility criteria they were invited to return to the ED for infusion.

To meet eligibility requirements for bamlanivimab infusion, patients must have had fewer than 10 days of symptoms, mild or moderate disease with no oxygen requirement, and were considered high risk for progression to severe disease based on the following criteria:

- ≥65 years of age or
- ≥55 years of age AND with one of the following:
  - Cardiovascular disease, hypertension, or chronic obstructive pulmonary disease/other chronic respiratory disease such as asthma.
- Body mass index (BMI) ≥35
- Chronic kidney disease
- Diabetes mellitus type 2
- Immunosuppressive disease or taking immunosuppressive medication.

### Exclusion

Excluded from the study were patients who did not consent for treatment, pregnant women, patients who did not meet the above inclusion criteria, and patients who upon initial presentation already had an oxygen requirement or required immediate admission to the hospital.

### Study Setting

As part of the response to the large influx of COVID-19 positive patients, ECRMC erected an emergent, tent-based COVID-19 hospital and monoclonal antibody infusion center in the ED parking lot where patients were treated from November 2020–January 2021. Eligible patients diagnosed with SARS-CoV-2 infection were either referred by their primary care physician within the 10-day symptom onset for monoclonal antibody infusions or were diagnosed at our COVID-19 tent hospital within the same time frame. During this surge, the county health department initially allocated a very limited supply of bamlanivimab for the hospital. Bamlanivimab was at times randomly distributed due to demand exceeding supply on a given day. The ECRMC pharmacists were also required to verify medications on site and prepare the monoclonal antibody infusions during business hours only. Therefore, bamlanivimab was available on a first-come first-served basis during business hours.

### Procedure

Emergency department patients were consented and given information regarding bamlanivimab and the risks and benefits of treatment in their preferred language, which was primarily Spanish. The treatment was unblinded to the patient and the associated healthcare workers. Patients in the treatment group received bamlanivimab (700 milligrams per 20 milliliters [mL]) mixed with 250 mL normal saline and infused over one hour. After approval by ECRMC hospital pharmacist, an infusion would be sent to the ED. An observation time of one hour was performed by the patient’s nurse and physician following infusion to observe for any serious hypersensitivity reaction and anaphylaxis. If patients tolerated medication and had no adverse response after one hour they were discharged from the ED.

### Data Collection

We performed a retrospective chart review from November 1, 2020–January 31, 2021 of patients diagnosed with mild to moderate SARS-CoV-2 infection who presented to ECRMC ED or were referred from community primary care physicians. A convenience sample consisted of eligible patients who received treatment with bamlanivimab (exposed patients) and eligible unexposed patients who tested positive for COVID-19 in the ED during this time same period. We collected data directly through a retrospective review of the hospital’s EHR. We then performed a search of the patient’s chief complaint and the *International Classification of Diseases, 10th revision*, billing codes in the ED EHR using MedHost information management software (MedHost, Inc., Franklin, TN), and we reviewed documentation by the treating emergency clinician and the pharmacy infusion records. Patient characteristics from both exposed group and unexposed group were recorded on a password-protected, patient-deidentified Microsoft Excel spreadsheet (Microsoft Corporation, Redmond, WA). These specific characteristics included age, gender, BMI, ethnicity, and comorbidities including cardiovascular disease/hyperlipidemia, hypertension, chronic obstructive pulmonary disease (COPD) or other chronic respiratory disease such as asthma, chronic kidney disease, diabetes mellitus, or current disease state of immunosuppression or currently taking immunosuppressive medication (eg, immunotherapy, anticancer drugs, etc.) and reported COVID-19 symptom onset (days).

Using Cerner healthcare technology services (Cerner, North Kansas City, MO), we assessed patient outcomes at 14 days post treatment through health records for inpatient care and for return visits. We additionally reviewed severe COVID-19 complications including repeat ED visits, hospitalization, transfer to outside hospitals, and complications such as intubation and death. We performed follow up on these patients with San Diego Health Connect (San Diego, CA) to ensure that the patients had not beem seen or admitted at a different county hospital.

### Statistical Analysis

We used frequencies and percentages to express categorical data such as ethnicity, gender, age ≥ 55 years or older, BMI ≥ 35, and the presence of at least one comorbidity (coronary artery disease, hypertension, diabetes, cancer/lymphoma, current use of immunosuppressive drug therapy, chronic kidney disease/dialysis, or chronic respiratory disease). Additionally, these factors (variables) along with the exposure to bamlanivimab or absence of exposure were examined to see whether there were associations with the following outcomes within 14 days: ED visit, hospitalization, and mortality.

Means and standard deviations were used to express continuous data such as age, BMI, and time since onset of COVID-19 symptoms. We used t-tests to examine whether there was a statistically significant difference between the unexposed groups and the bamlanivimab group. Chi-square tests and bivariate analyses were used to find an association between the exposure to bamlanivimab and patients’ characteristics and outcomes variables. Using bivariate analysis, factors that were significant to α level of 0.10 were then entered in the full multivariate regression models for ED return visits in 14 days and hospitalizations. The factors that were significant to α level of 0.05 or less were then kept and entered into the final, reduced multivariate regression models. We used the variables that reached to α level of 0.05 in the final, reduced models to calculate reduction of the outcome – ED return visits in 14 days and hospitalizations. All analyses were performed using SAS© Studio Release 3.8 (Cary, North Carolina, USA).

## RESULTS

The ECRMC ED with an annual volume of 46,000 patients had a total of 7,735 patients within the three-month period, November 1, 2020–January 31, 2021. To detect a 15% different in treatment effect and using a confidence level of 95%, α of 0.05, and 80% power, we determined that a total sample size would need to be 256; at least 57 patients per treatment arm were required for the likelihood ratio chi-square for the outcome ED return visits. For the same parameters, 87 hospitalized patients were required.^17^ We reviewed records from a total of 276 COVID-19 positive patients from the ED. Six patients were excluded due to incomplete health records. A total of 270 patient records were included, 136 patients in the unexposed group and 134 patients in the bamlanivimab arm. The demographics, characteristics, and comorbidities of interest data are summarized in Table 1. The two groups were comparable for age, gender, ethnicity, and BMI. However, the bamlanivimab patients were more likely than unexposed group to have the presence of one or more comorbidities (*P*<.0001), age ≥ 55 years old (*P* <.0001), and BMI ≥ 35 (*P* = 0.0032). Overall, 80.7% patients reported at least one of the comorbidities. The frequencies of each of the comorbidities in both the unexposed and exposed groups are summarized in Table 1. There was a statistically significant difference in the proportion of patients with chronic respiratory disease (*P* = 0.0094) and cancer (*P* = 0.0422) within the exposed group being higher.

Outcomes of both unexposed patients, exposed bamlanivimab patients, and the differences in their proportions between groups are reported in Table 2.

Using an α of 0.05 for significance, we found there was a statistically significant difference between the two groups in the 14-day outcomes of return visit to the ED (*P* < .0001), hospitalization (*P* = 0.0011), and death (*P* = 0.0235). In building the multivariate regression models of outcomes of ED visits within 14 days (Appendix i) and subsequent hospitalizations (Appendix ii), we used bivariate associations from the patient demographics, comorbidities, and exposure to bamlanivimab infusions. Due to lack of data on the outcomes of ventilator requirement and death, the variables (factors) and bamlanivimab exposure could not be analyzed through regression modeling (Appendix iii).

### Outcomes

Overall, the unstratified unexposed patient had a 2.00 (95% confidence interval [CI]: 1.340, 2.977) increased risk of an ED return visit in 14 days and a 2.27 (95% CI: 1.224, 4.208) increased risk of hospitalization compared to those who received bamlanivimab (exposed group). There were no deaths in the exposed group and five deaths overall in the unexposed group. We placed the variables into a bivariate analysis to build various regression models of ED return visits in 14 days and hospitalizations (Appendix iv–ix). The bivariate associations that were used in producing the full models were made by removing those variables that did not reach α level of 0.10. These variables are subsequently placed in intermediate regression models (Appendix iv–ix). The final outcome, reduced model, was then produced and in which variables were found to be significant at an α level of 0.05. The final, reduced multivariate regression outcome models showed that those exposed to bamlanivimab was a significant contributor to decreased ED return visits in 14 days and subsequent hospitalizations (Table 3 and Table 4).

The final regression models show that the exposed group (bamlanivimab) had a 22.8% (mean estimate = 0.7717, 95% CI: 0.6482, 0.8611) risk reduction or 84.4% (0.3030, 95% CI: 0.166, 0.554, *P* =.0001) absolute reduction of ED return visits within 14 days compared to the unexposed groups after adjusting for chronic kidney disease (Table 4). The bamlanivimab group had 19.0% (mean estimate = 0.8097, 95% CI: 0.6451, 0.9087) risk reduction or 96.2% (0.235, 95% CI: 0.100, 0.550, *P* = 0.0008) absolute reduction of subsequent hospitalizations compared to unexposed patients after adjusting for diabetes status (Table 4).

Adverse drug reactions to bamlanivimab were recorded and all nine reports were minor. Post-infusion, there were two patients with nausea/vomiting, one patient with worsening dyspnea, five patients with elevated temperature (> 0.6°C), two patients with chest pain, and no patients with allergic/anaphylaxis reaction. Although all patients were initially discharged from the ED after their infusion of bamlanivimab, four of the nine patients with adverse reactions were later hospitalized due to worsening of COVID-19 pneumonia. There were no subsequent deaths reported in the nine patients who reported an adverse drug reaction.

The number needed to treat for ED visits within 14 days was 4.73 and for hospitalizations was 7.61. The number needed to harm for ED visits within 14 days was 0.047 and for subsequent hospitalizations was 0.076.

## DISCUSSION

The data on monoclonal antibody therapies (casirivimab/imdevimab and bamlanivimab) that were given an EUA by the FDA in November 2020 is constantly evolving. While the majority of the studies to date show a reduction of viral load, there are some studies on patient-centered outcomes and the impact of initiating therapeutic regimens on hospital systems.^11^,^14^,^18^ Previous data demonstrated a 7% absolute risk reduction for hospitalization and viral load reduction in patients receiving early monoclonal antibody infusion.^10^ Recently, a randomized control trial of high-risk residents in US skilled nursing facilities who received bamlanivimab demonstrated decreased incidence of SARS-CoV-2 infections at eight weeks.^19^

Due to variants, the FDA in April 2021 issued a statement that bamlanivimab cannot be used as a single-therapy regimen for mild-moderate COVID-19 in high-risk patients. Therefore, bamlanivimab was being combined with etesevimab until June 25, 2021, when the EUA was then discontinued for the combination.^20^–^22^ However, a few studies have focused on outcomes from EDs.^23^,^24^ One ED study demonstrated that there was no significant difference in comparing a single-therapy regimen of bamlanivimab vs a combination of casirivimab/imdevimab on hospitalization outcomes.^23^ A smaller observational ED study demonstrated that 78% (45) of their eligible patients given bamlanivimab were discharged from the ED and 14% (8) were hospitalized.^24^

Even prior to the COVID-19 pandemic, ED crowding has been a major issue faced by EDs throughout the US. In the ECRMC ED, which already had the highest patient-to-bed ratio of any ED in San Diego or Imperial counties prior to the pandemic, crowding reached critical levels.^25^ During the November surge, the number of inpatients at ECRMC was nearly double the normal census. Regional referral centers, overburdened by their own COVID surges, stopped accepting transfers, making it difficult to reduce the burden on ECRMC by transferring patients out of county. Critically ill patients often remained in the ED for days at a time.

Our study is one of the first to date to examine the efficacy of administering a monoclonal antibody to a primarily Latinx/Hispanic population in an ED at a border hospital, specifically to mitigate COVID-19 hospitalizations. Our regression modeling demonstrated significant reduction of return visits to the ED and in hospitalizations after controlling for chronic kidney disease and diabetes, respectively. These significant reductions may impact mortality due to the decrease in ED return visits and hospitalizations.

The original bamlanivimab study used for the FDA EUA showed that the overall incidence of ED return visit, hospitalization, and/or death within 30 days was only 5.8% in the placebo group.^14^ Even the high-risk patients in the placebo group of this study (those 65 years and older or with BMI ≥ 35) only had hospitalization rates of 13.5%. However, less than half of the patients in that study were Latinx/Hispanic, a disadvantaged group known to have a higher incidence of hospitalization and death from COVID-19.^26^ Not only was our population predominantly Latinx/Hispanic, they also had a high incidence of risk factors for complications from COVID-19. Therefore, monoclonal infusions may have had a much more pronounced impact on disease reduction in these populations, and it is significant that we saw such a substantial decrease in return visits and hospitalizations in our unique population.

Financially, the costs of monoclonal antibody infusion are substantially less compared to hospitalization for COVID-19. According to the Centers for Medicare and Medicaid Services, the cost of hospitalization for COVID-19 has been estimated to be from $21,936 to $74,310 depending on severity, complications, and insurance coverage.^27^ The cost of the drug itself is zero, but the costs incurred from infusion of monoclonal antibodies are between $350 – $750.^28^

## LIMITATIONS

This was an observational study conducted in a single-center ED at a US-Mexico border hospital; thus, it may not be generalizable to the larger population. Additionally, selection bias may have been introduced through the non-randomized convenience sampling of patients with multiple comorbidities and mild symptoms of SARS-CoV-2 infection. Because the researchers were aware of the treatment allocation, this could have also biased their assessment.

The distribution of bamlanivimab was unpredictable due to the surging prevalence of COVID-19 in the community. This created a lack of predictability in who ultimately received treatment with bamlanivimab, and eligible patients may or may not have received treatment in a timely manner. However, this may have been minimized by an ongoing active campaign to inform patients and physicians within the community regarding bamlanivimab infusions in the ED such that patients did come during designated hours of infusion, which would have enabled us to include as many potential eligible patients as possible. Although some may question the reliability of the ED return visit outcome, the patients in our study were followed in detail and the data appears to support a substantial decrease in preventing subsequent hospitalizations for the exposed group. Additionally, the endotracheal intubation/ventilator and mortality outcomes were too small to allow for comparisons. These outcomes were likely confounded because some patients who expired had been placed on comfort care prior to being intubated.

Given concerns that COVID-19 surges are imminent, we decided that these results should be submitted for publication in hopes that the data could be used to quickly operationalize in areas most vulnerable to overwhelming COVID-19 surges. It is noteworthy that the November and December surge was prior to the discovery of the several COVID-19 variants now known to be spreading across the country. There is evidence to suggest that bamlanivimab alone may not have similar effectiveness against variants.^29^ This may be likely due to the decreased predominance of more virulent and/or transmissible variants that are not susceptible to neutralization and the rise of these variants of concern that can escape neutralization of monoclonal antibody treatment. Therefore, FDA protocols are now for other combination monoclonal antibody treatment.^21^–^23^

Future studies may focus on how monoclonal antibody infusions could efficiently be operationalized as these ED visits could potentially increase the number of patients seeking monoclonal antibody treatment, which could be a potential confounder in surges. Additionally of value would be a multicenter, randomized, and placebo-controlled study with a large proportion of patients who belong to vulnerable populations such as Latinx/Hispanics in order to observe whether or not monoclonal antibodies do have a similar effect, especially with the advent of variants of SARS-CoV-2.

## CONCLUSION

Our study found a 22.8% risk reduction in return ED visits and 19.0% risk reduction of subsequent hospitalizations in high-risk COVID-19 patients who received bamlanivimab compared to those who did not receive bamlanivimab. We believe that operationalizing monoclonal antibody infusions in high-risk COVID-19 patients could be made into an effective strategy for mitigating the COVID-19 surges at lower-resourced US-Mexico border hospitals.

## Supplementary Information





## Figures and Tables

**Table 1:** Demographics and characteristics of COVID-19 emergency department patients, comparing no bamlanivimab exposure vs bamlanivimab exposure.

Characteristics	All	No bamlanivimab (unexposed)	bamlanivimab (exposed)	*P*-value
Age				
Mean (SD)	61.7 (13.6)	63.3 (12.4)	60.3 (14.7)	
Median	62.0	63.0	62.0	0.0681
Min, max	19, 93	20, 93	19, 91	
BMI				
Mean (SD)	31.0 (6.6)	30.2 (4.9)	31.8 (7.9)	
Median	29.4	29.4	29.4	0.0517
Min, max	17.1, 61.1	17.1, 45.6	21.0, 61.1	
Symptom onset (days)^b^				
Mean (SD)	4.9 (4.0)	5.2 (4.5)	4.6 (3.3)	
Median	4.0	4.0	4.0	0.2144
Min, max	1.0, 28.0	1.0, 28.0	1.0,18.0	
	*No. (%)*	*No. (%)*	*No. (%)*	
	N = 270^a^	N = 136	N = 134	
Ethnicity				
Latinx/Hispanic	247 (91.5)	128 (94.1)	119 (88.8)	0.1180
Other^d^	23 (8.5)	8 (5.88)	15 (11.2)	
Gender				
Male	140 (51.9)	71 (52.2)	69 (51.5)	0.9066
Female	130 (48.1)	65 (47.8)	65 (48.5)	
Age ≥ 55 years old	209 (77.4)	120 (88.2)	89 (66.4)	<.0001
	N = 257	N = 128	N = 129	
BMI ≥ 35^c^	58 (22.6)	19 (14.8)	30 (30.2)	0.0032
Missing	13	8	5	
	N=270^a^	N=136	N=134	
Comorbidities^e^	218 (80.7)	94 (69.1)	124 (92.5)	<.0001
CAD/HLD^f^	50 (18.5)	24 (17.6)	26 (19.4)	0.7104
HTN	161 (59.6)	77 (56.6)	84 (62.7)	0.3095
DM	111 (41.1)	48 (35.3)	63 (47.0)	0.0503
CKD^g^	12 (4.4)	6 (4.48)	6 (4.41)	0.9791
Immunocompromised^h^	17 (6.3)	6 (4.41)	11 (8.21)	0.1990
Cancer	16 (5.9)	12 (8.82)	4 (3.00)	0.0422
CRD^i^	29 (10.7)	8 (5.9)	21 (15.7)	0.0094

a Column percentages are represented of the total N = 270, unexposed patients N = 136, exposed patients N = 134, unless the data is specified as missing.

b Patient reported symptom onset of COVID-19 during evaluation in the ED.

c Body mass index (BMI) missing for 13 patients. Total N = 257, unexposed patients N = 128, exposed patients N = 129.

d Other ethnicity/race who identified themselves as White, Black, or Asian, or non-Latinx/Hispanic.

e At least one of the listed comorbidities: diabetes (DM), coronary artery disease/hyperlipidemia (CAD/HLD), hypertension (HTN), chronic kidney disease (CKD), chronic respiratory disease (CRD), immunosuppression, cancer/lymphoma (Cancer).

f CAD/HLD - History/documented cardiac stents, coronary artery bypass surgery, hyperlipidemia on lipid-lowering agents.

*SD*, standard deviation; *COVID-19*, coronavirus disease 2019.

g History/documented renal failure, peritoneal or hemodialysis.

h Current immunosuppressive therapy such as steroids, anti-cancer, protein drugs, among others.

i CRD includes asthma, pulmonary fibrosis, or chronic obstructive pulmonary disease (COPD), among other chronic lung diseases

**Table 2:** Clinical outcomes of COVID-19 patients no bamlanivimab exposure vs bamlanivimab exposure.

Outcomes^a^	All patients	No Bamlanivimab (Unexposed)	Bamlanivimab (Exposed)	*P*-value
	N=270 *No. (%)*	N = 136 *No. (%)*	N = 134 *No. (%)*	
Return visit to ED in 14 days	67 (24.8)	48 (35.3)	19 (14.2)	<0.0001
Hospitalization in 14 days	34 (12.6)	26 (19.1)	8 (6.0)	0.0011
Endotracheal intubation^b^,^d^	3 (1.13)	3 (2.22)	0 (0)	0.0862
Missing	4	4	0	
Mortality^c^,^d^				0.0235
Survived	262 (98.2)	128 (96.2)	134 (100.0)	
Died	5 (1.9)	5 (3.76)	0 (0.0)	
Missing	3	3	0	

a Column percents presented.

b Endotracheal intubation data missing from 4 patients. Total N = 266, unexposed patients N = 132, exposed patients N = 134.

c Mortality data is missing outcomes of three patients, with total N = 267, unexposed patients N = 132, exposed patients N = 134.

d Chi-square analysis is unreliable due to >25% of the data missing in cells.

*ED*, emergency department.

**Table 3:** Intermediary regression outcome models for COVID-19 ED return visits in 14 days and hospitalizations.

Outcome	Variable N = 270^a^	Estimate	Standard error	Likelihood ratio 95% CI	Wald Chi-Square	*P*-value
ED return visits within 14 days						
	Bamlanivimab	−1.24	0.377	−1.220, −1.220	10.8	0.0010^b^
	Male	0.464	0.324	0.464, 0.4634	2.05	0.152
	Age ≥ 55^b^	0.915	0.476	0.915, 0.915	3.69	0.0546^b^
	CKD^b^	1.184	0.701	1.18, 1.18	2.85	0.0914^b^
	Bamlanivimab	−1.34	0.4982	−2.39, −0.412	7.24	0.0071^b^
	Male	0.908	0.440	0.0677, 1.808	4.25	0.0391^b^
	Comorbidities	0.4859	0.7869	−1.045, 2.092	0.38	0.5370
Hospitalizations						
	Age ≥ 55	1.28	0.810	−0.138, 3.20	2.48	0.115^c^
	CAD/HLD	−0.3522	0.5407	−1.496, 0.654	0.42	0.5149
	DM	0.686	0.464	−0.205, 1.63	2.18	0.140^c^
	HTN	−0.4090	0.5446	−1.459,0.7025	0.56	0.4527
	CRD	0.2895	0.6736	−1.145, 1.555	0.18	0.6673
	Immunosuppressed	−0.1616	0.8893	−2.099, 1.457	0.03	0.8558
	Cancer	1.13	0.743	−0.370, 2.60	2.30	0.129^c^
	CKD	0.3769	0.8404	−1.422, 1.947	0.20	0.6538

a No data missing.

b Variables were statistically significant at a α =0.1 in bivariate analysis and were included in the full regression model.

C Variables that were significant in the bivariate analysis at a α =0.1, but not found to be statistically significant and were re-entered into the developing models prior to the final reduced model.

*CI*, confidence interval; *ED*, emergency department; *CKD*, chronic kidney disease; *CAD*, coronary artery disease; *HLD*, hyperlipidemia; *DM*, diabetes mellitus; *HTN*, hypertension; *CRD*, chronic respiratory disease.

**Table 4:** Final, reduced multivariate regression outcome models of ED visits within 14 days and subsequent hospitalizations.

Outcome	Variable N = 270^a^	Estimate	Standard error	Likelihood ratio 95% CI	Wald Chi-Square	P-value
ED return visits within 14 days						
	Bamlanivimab	−1.22	0.31	−0.63, −1.84	15.48	<.0001^b^
	CKD	1.28	0.63	0.297, 2.53	4.16	0.0413^b^
Hospitalizations						
	Bamlanivimab	−1.45	0.4339	− 2.36, −0.639	11.14	0.0008^b^
	DM	0.8854	0.38	1.65, 0.1372	5.30	0.0213^b^

a No data missing.

b Variables that reached statistical significance at α =0.05 in the final models.

Those receiving bamlanivimab on average had 22.83% (mean estimate = 0.7717; CI: 0.6482, 0.8611) less risk of having an ED return visit in 14 days after adjusting for CKD status (*P* <0.0001).

Those receiving bamlanivimab on average had 19.03% (mean estimate = 0.8097, CI: 0.6451, 0.9087) less risk of being hospitalized after adjusting for diabetic status (*P* = 0.0008).

*ED*, emergency department; *CKD*, chronic kidney disease; *DM*, diabetes mellitus.
