# Supplementary material for: Bamlanivimab Reduces ED Returns and Hospitalizations and May Reduce COVID-19 Burden on Low-resource Border Hospitals
Source: West J Emerg Med. 2022 Mar 17;23(3):302–11. doi: 10.5811/westjem.2021.10.52668 (PMC9183783; doi:10.5811/westjem.2021.10.52668)
Supplement: Supplementary file 1 [file wjem-23-302-s001.docx]

| **Table 1. Demographics and Characteristics of SARS-CoV-2 positive Emergency Department Patients** | | | | |
| --- | --- | --- | --- | --- |
| **Characteristics (n=270)** | **All**  **n (%)** | **Control**  **(n %)** | **Monoclonal Antibody**  **(n%)** | **p-value** |
| Age |  |  |  |  |
| Mean (SD) | 61.7 (13.6) | 63.3 (12.4) | 60.3 (14.7) |  |
| Median | 62.0 | 63.0 | 62.0 | 0.0681 |
| Min, Max | (19, 93) | (20, 93) | (19, 91) |  |
| BMI |  |  |  |  |
| Mean (SD) | 31.0 (6.6) | 30.2 (4.9) | 31.8 (7.9) |  |
| Median | 29.4 | 29.4 | 29.4 | 0.0517 |
| Min, Max | (17.1, 61.1) | (17.1, 45.6) | (21.0, 61.1) |  |
| Symptom onset ED visit (Days)* |  |  |  |  |
| Mean (SD) | 4.9 (4.0) | 5.2 (4.5) | 4.6 (3.3) | 0.2144 |
| Median | 4.0 | 4.0 | 4.0 |  |
| Min, Max | (1.0,28.0) | (1.0, 28.0) | (1.0,18.0) |  |
|  |  |  |  |  |
| Ethnicity |  |  |  |  |
| Latinx/Hispanic | 247 (91.5) | 128 (51.8) | 119 (48.2) | 0.1180 |
| Other** | 23 (8.5) | 8 (34.8) | 15 (65.2) |  |
| Gender |  |  |  |  |
| Male | 140 (51.9) | 71 (50.7) | 69 (49.3) | 0.9056 |
| Female | 130 (48.2) | 65 (50.0) | 65 (50.0) |  |
| Comorbidities*** |  |  |  |  |
| Yes | 218 (80.7) | 94 (43.1) | 124 (56.9) | <.0001 |
| No | 52 (19.3) | 42 (80.8) | 10 (19.2) |  |
| Age > 55 |  |  |  |  |
| Yes | 209 (77.4) | 120 (57.4) | 89 (42.6) | <.0001 |
| No | 61 (22.6) | 16 (26.2) | 45 (73.8) |  |
| BMI > 35 |  |  |  |  |
| Yes | 58 (22.6) | 19 (32.8) | 39 (67.2) | 0.0032 |
| No | 199 (77.4) | 109 (54.8) | 90 (45.2) |  |
| ^a^ Missing data from 6 patients total  ^b^ Other- Ethnicity/Race who identified themselves as White, Black, or Asian and non-Latinx/Hispanic  ^c^ Comorbidities – at least one of the listed comorbidities. | | | |  |

**Table 2. Detailed Comorbidities Emergency Department Patients**

|  | **All** | | **Control** | **Monoclonal Antibody** | **p-value** |
| --- | --- | --- | --- | --- | --- |
| Coronary Artery Disease/Hyperlipidemia* | |  |  |  |  |
| Yes | | 50 (18.5) | 24 (48.0) | 26 (52.0) | 0.7104 |
| No | | 220 (81.5) | 112 (50.9) | 108 (49.1) |  |
| Hypertension | |  |  |  |  |
| Yes | | 161 (59.6) | 77 (47.8) | 84 (52.2) | 0.3095 |
| No | | 109 (40.4) | 59 (54.1) | 50 (45.9) |  |
| Diabetic | |  |  |  |  |
| Yes | | 111 (41.1) | 48 (43.2) | 63 (56.8) | 0.0503 |
| No | | 159 (58.9) | 88 (55.4) | 71 (44.7) |  |
| Chronic Kidney Disease^+^ | |  |  |  |  |
| Yes | | 12 (4.4) | 6 (50.0) | 6 (50.0) | 0.9791 |
| No | | 258 (95.6) | 130 (50.4) | 128 (49.6) |  |
| Immunocompromise^++^ | |  |  |  |  |
| Yes | | 17 (6.3) | 6 (35.3) | 11 (64.7) | 0.1990 |
| No | | 253 (93.7) | 130 (51.4) | 123 (48.6) |  |
| Cancer | |  |  |  |  |
| Yes | | 16 (5.9) | 12 (75.0) | 4 (25.0) | 0.0422 |
| No | | 254 (94.1) | 124 (48.8) | 130 (51.2) |  |
| Chronic Respiratory Disease^+++^ | |  |  |  |  |
| Yes | | 29 (10.7) | 8 (27.6) | 21 (72.4) | 0.0094 |
| No | | 241 (89.3) | 128 (53.1) | 113 (46.9) |  |

*Coronary Artery Disease – history/documented cardiac stents, coronary artery bypass surgery, hyperlipidemia on lipid-lowering agents.

+ Chronic Kidney Disease – history/documented renal failure, peritoneal or hemodialysis.

++Immunocompromise – currently on immunosuppressive therapy such as steroids, anti-cancer, protein drugs, among others.

+++Chronic Respiratory Disease – asthma, pulmonary fibrosis, or chronic obstructive pulmonary disease (COPD)

**Table 3. Outcomes SARS-CoV-2 Emergency Department patients**

|  | **All** | **Control** | **Monoclonal Antibody** | **P-value** |
| --- | --- | --- | --- | --- |
| Return Visit to ED in 14 days |  |  |  | <0.0001 |
| Yes | 67 (24.8) | 48 (71.6) | 19 (28.4) |  |
| No | 203 (75.2) | 88 (43.4) | 115 (56.7) |  |
| Hospitalization in 14 days |  |  |  | 0.0011 |
| Yes | 34 (12.6) | 26 (76.5) | 8 (23.5) |  |
| No | 236 (87.4) | 110 (46.6) | 126 (53.4) |  |
| Ventilator |  |  |  | 0.0862 |
| Yes | 3 (1.1) | 3 (100.0) | 0 (0.0) |  |
| No | 263 (98.9) | 132 (50.2) | 131 (49.8) |  |
| Missing | 4 |  |  |  |
| Died |  |  |  | 0.0235 |
| Yes | 5 (1.9) | 5 (100.0) | 0 (0.0) |  |
| No | 262 (98.2) | 128 (48.9) | 134 (51.2) |  |
| Missing** | 3 |  |  |  |
| Improved |  |  |  | 0.1565 |
| Yes | 222 (82.5) | 107 (48.2) | 115 (51.8) |  |
| No | 47 (17.5) | 28 (59.6) | 19 (40.4) |  |
| Missing | 1 |  |  |  |

**Patients were transferred to outside hospitals and therefore records could not be obtained regarding their care.

**Table 4. Full Regression Model for ED Return Visits in 14 days**

| **Variable** | ***ED Return Visits in 14 days*** | | | | | |
| --- | --- | --- | --- | --- | --- | --- |
|  | **DF** | **Estimate** | **Standard Error** | **Likelihood Ratio 95 % CL** | **Wald Chi-Square** | **P-Value** |
| Bamlanivimab | 1 | -1.058 | 0.33 | -1.06, -1.06 | 10.51 | 0.0012^**^ |
| Age > 55 | 1 | 0.494 | 0.43 | 0.49, 0.49 | 1.32 | 0.2509 |
| Intubated | 1 | 23.762 | 66518.59 | 23.76, 23.76 | 0.00 | 0.9997 |
| CKD | 1 | 1.133 | 0.65 | 1.13, 1.13 | 3.00 | 0.0834^*^ |
| *The full model includes all variables significant at an alpha of 0.10 in the bivariate analysis.*  *Those receiving Bamlanivimab on average had 74.24% (mean estimate = 0.2576, CI [0.1547, 0.3968]) less risk of having an ED return visit in 14 days after adjusting for all other variables in the mode (p = 0.0012).*  ^**^Significant at an alpha of 0.05.  ^*^Significant at an alpha of 0.10. | | | | | | |

**Table 5. Full Logistic Regression Model of Hospitalizations**

| **Variable** | ***Hospitalizations*** | | | | | |
| --- | --- | --- | --- | --- | --- | --- |
|  | **DF** | **Estimate** | **Standard Error** | **Likelihood Ratio 95 % CL** | **Wald Chi-Square** | **P-Value** |
| Bamlanivimab | 1 | -1.07 | 0.45 | -2.01, 0.22 | 5.61 | 0.0178^**^ |
| Male | 1 | 0.73 | 0.41 | -0.06, 1.57 | 3.15 | 0.0762^*^ |
| Age > 55 | 1 | 1.10 | 0.77 | -0.21, 2.98 | 2.04 | 0.1531 |
| DM | 1 | 0.08 | 0.40 | 0.02, 1.62 | 4.02 | 0.0450^**^ |
| Cancer | 1 | 0.99 | 0.67 | -0.42, 2.25 | 2.20 | 0.1380 |
| *The full model includes all variables significant at an alpha of 0.10 in the bivariate analysis.*  *Those receiving Bamlanivimab on average had 74.46% (mean estimate = 0.2554, CI [0.1240, 0.4539) less risk of being hospitalized after adjusting for all other variables in the mode (p = 0.00178).*  ^**^Significant at an alpha of 0.05.  ^*^Significant at an alpha of 0.10. | | | | | | |

**Table 6. Full Logistic Regression Model of Mortality**

| **Variable** | **Mortality** | | | | | |
| --- | --- | --- | --- | --- | --- | --- |
|  |  | | | | | |
|  | **DF** | **Estimate** | **Standard Error** | **Likelihood Ratio 95 % CL** | **Wald Chi-Square** | **P-Value** |
| Bamlanivimab | 1 | -25.83 | 202014.80 | -25.83, -25.83 | 0.00 | 0.9999 |
| Cancer | 1 | 1.96 | 1.03 | 1.96, 1.96 | 3.65 | 0.0562^*^ |
| CKD | 1 | 1.20 | 1.33 | 1.20, 1.20 | 0.81 | 0.3680 |
| *The full model includes all variables significant at an alpha of 0.10 in the bivariate analysis.*  *There were no patients in the Bamlanivimab group who died.*  ^*^Significant at an alpha of 0.10. | | | | | | |

**Table 7. Final Regression Model of ED Visits within 14 days**

| **Variable** | **ED Return Visits in 14 days** | | | | | |
| --- | --- | --- | --- | --- | --- | --- |
|  |  | | | | | |
|  | **DF** | **Estimate** | **Standard Error** | **Likelihood Ratio 95 % CL** | **Wald Chi-Square** | **P-Value** |
| Bamlanivimab | 1 | 1.22 | 0.31 | 0.63, 1.84 | 15.48 | <0.0001^**^ |
| CKD | 1 | -1.28 | 0.63 | -2.53, -0.03 | 4.16 | 0.0413^**^ |
| *The reduced model includes all variables significant at an alpha of 0.05.*  *Those receiving Bamlanivimab on average had 22.83% (mean estimate = 0.7717, CI [0.6482, 0.8611]) less risk of having an ED return visit in 14 days after adjusting for CKD status (p <0.0001 ).*  ^**^Significant at an alpha of 0.05. | | | | | | |

**Table 8. Final Regression Model of Hospitalizations**

| **Variable** | **Hospitalizations** | | | | | |
| --- | --- | --- | --- | --- | --- | --- |
|  |  | | | | | |
|  | **DF** | **Estimate** | **Standard Error** | **Likelihood Ratio 95 % CL** | **Wald Chi-Square** | **P-Value** |
| Bamlanivimab | 1 | 1.45 | 0.43 | 0.64, 2.36 | 11.14 | 0.0008^**^ |
| DM | 1 | -0.89 | 0.38 | -1.65, -0.14 | 5.30 | 0.0213^**^ |
| *The reduced model includes all variables significant at an alpha of 0.05.*  *Those receiving Bamlanivimab on average had 19.03% (mean estimate=0.8097, CI [0.6451, 0.9087]) less risk of being hospitalized after adjusting for diabetic status (p =0.0008 ).*  ^**^Significant at an alpha of 0.05. | | | | | | |

**Table. Final Regression Model of Mortality**

| **Variable** | ***Mortality*** | | | | | |
| --- | --- | --- | --- | --- | --- | --- |
|  | **DF** | **Estimate** | **Standard Error** | **Likelihood Ratio 95 % CL** | **Wald Chi-Square** | **P-Value** |
| Bamlanivimab | 1 | -26.11 | 20317.8 | -26.11, -26.11 | 0.00 | 0.9999 |
| CKD | 1 | 1.82 | 1.21 | 1.82, 1.82 | 2.26 | 0.1325 |
| *The reduced model includes all variables significant at an alpha of 0.05, as well as the treatment arm (not significant at alpha = 0.05).*  *There were no mortalities in the Bamlanivimab group.* | | | | | | |

| **Appendix i. Bivariate Associations ED Return Visits**  **Table** | | **Non-Stratified**  **Bivariate Associations** | | | **Control**  **Bivariate Associations** | | | **Monoclonal Antibody**  **Bivariate Associations** | | | | |
| --- | --- | --- | --- | --- | --- | --- | --- | --- | --- | --- | --- | --- |
|  |  | **Return ED Visit, No. (%)**  **(*n*=136)** | **OR (95% CI)** | ***P*** | **Return ED Visit, No. (%)**  **(*n=*)** | **OR (95% CI)** | ***P*** | **Return ED Visit, No. (%)**  **(*n*=134)** | **OR (95% CI)** | | ***P*** |  |
| Ethnicity |  | 0.0040 | | |  | 0.0313 | |  | 0.0948 | | | |
| Latino/Hispanic | | 67 (24.8) | -- | | 48 (35.3) | -- | | 19 (14.2) | -- | | |  |
| Other | | 0 (0.0) | 1.00 (Referent) | | 0 (0.0) | 1.00 (Referent) | | 0 (0.0) | 1.00 (Referent) | | |  |
| Gender | | | 0.3581 | |  | 0.2907 | |  | 0.9146 | | | |
| Male | | 38 (14.1) | 1.30 (0.74, 2.26) | | 28 (20.6) | 1.47 (0.72, 2.98) | | 10 (7.5) | 1.05 (0.40, 2.79) | | |  |
| Female | | 29 (10.7) | 1.00 (Referent) | | 20 (14.7) | 1.00 (Referent) | | 9 (6.7) | 1.00 (Referent) | | |  |
| Age > 55 | | | 0.0162 | |  | 0.0422 | |  | 0.8418 | | | |
| Yes | | 59 (21.9) | 2.61 (1.17, 5.81) | | 46 (33.8) | 4.35 (0.95, 20.03) | | 13 (9.7) | 1.11 (0.39, 3.15) | | |  |
| No | | 8 (3.0) | 1.00 (Referent) | | 2 (1.5) | 1.00 (Referent) | | 6 (4.5) | 1.00 (Referent) | | |  |
| Comorbidities | |  | 0.9726 | |  | 0.2728 | |  | 0.6937 | | |  |
| Yes | | 54 (20.0) | 0.99 (0.49, 1.99) | | 36 (26.5) | 1.55 (0.71, 3.41) | | 18 (13.4) | 1.53 (0.18, 12.80) | | |  |
| No | | 13 (4.8) | 1.00 (Referent) | | 12 (8.8) | 1.00 (Referent) | | 1 (0.8) | 1.00 (Referent) | | |  |
| Obesity* | |  | 0.6184 | |  | 0.9290 | |  |  | 0.7575 | |  |
| Yes | | 13 (5.1) | 0.84 (0.42, 1.68) | | 7 (5.5) | 1.05 (0.38, 2.88) | | 6 (4.7) | 1.18 (0.41, 3.42) | | |  |
| No | | 51 (19.8) | 1.00 (Referent) | | 39 (30.5) | 1.00 (Referent) | | 12 (9.3) | 1.00 (Referent) | | |  |
| Diabetic | |  | 0.2020 | |  | 0.0575 | |  | 0.5965 | | |  |
| Yes | | 32 (11.9) | 1.44 (0.82, 2.50) | | 22 (16.2) | 2.02 (0.97, 4.18) | | 10 (7.5) | 1.30 (0.49, 3.44) | | |  |
| No | | 35 (13.0) | 1.00 (Referent) | | 26 (19.1) | 1.00 (Referent) | | 9 (6.7) | 1.00 (Referent) | | |  |
| Hypertension | |  | 0.7846 | |  | 0.1663 | |  | 0.0491 | | |  |
| Yes | | 39 (14.4) | 0.93 (0.53, 1.62) | | 31 (22.8) | 1.67 (0.81, 3.44) | | 8 (6.0) | 0.37 (0.14, 1.00) | | |  |
| No | | 28 (10.4) | 1.00 (Referent) | | 17 (12.5) | 1.00 (Referent) | | 11 (8.2) | 1.00 (Referent) | | |  |
| Coronary Artery Disease | |  | 0.3826 | |  | 0.8247 | |  | 0.2909 | | |  |
| Yes | | 10 (3.7) | 0.72 (0.34, 1.52) | | 8 (5.9) | 0.90 (0.35, 2.29) | | 2 (1.5) | 0.45 (0.10, 2.07) | | |  |
| No | | 57 (21.1) | 1.00 (Referent) | | 40 (29.4) | 1.00 (Referent) | | 17 (12.7) | 1.00 (Referent) | | |  |
| Chronic Kidney Disease | |  | 0.0388 | |  | 0.0118 | |  | 0.8582 | | |  |
| Yes | | 6 (2.2) | 3.23 (1.00, 10.38) | | 5 (3.7) | 10.12 (1.15, 89.30) | | 1 (0.8) | 1.22 (0.14, 11.08) | | |  |
| No | | 61 (22.6) | 1.00 (Referent) | | 43 (31.6) | 1.00 (Referent) | | 18 (13.4) | 1.00 (Referent) | | |  |
| Immunodeficiency | |  | 0.8991 | |  | 0.4407 | |  | 0.6136 | | |  |
| Yes | | 4 (1.5) | 0.93 (0.29, 2.95) | | 3 (2.2) | 1.89 (0.37, 9.73) | | 1 (0.8) | 0.58 (0.07, 4.84) | | |  |
| No | | 63 (23.3) | 1.00 (Referent) | | 45 (33.1) | 1.00 (Referent) | | 18 (13.4) | 1.00 (Referent) | | |  |
| Cancer | |  | 0.2258 | |  | 0.6285 | |  | 0.5288 | | |  |
| Yes | | 6 (2.2) | 1.90 (0.66, 5.44) | | 5 (3.7) | 1.35 (0.40, 4.49) | | 1 (0.8) | 2.07 (0.20, 21.05) | | |  |
| No | | 61 (22.6) | 1.00 (Referent) | | 43 (31.6) | 1.00 (Referent) | | 18 (13.4) | 1.00 (Referent) | | |  |
| Chronic Respiratory Disease | |  | 0.7146 | |  | 0.8929 | |  | 0.1683 | | |  |
| Yes | | 8 (3.0) | 1.18 (0.49, 2.79) | | 3 (2.2) | 1.11 (0.25, 4.85) | | 5 (3.7) | 2.21 (0.70, 6.98) | | |  |
| No | | 59 (21.9) | 1.00 (Referent) | | 45 (33.1) | 1.00 (Referent) | | 14 (10.5) | 1.00 (Referent) | | |  |
| **Obesity has 5 missing observations. P value derived from Chi-square. Odds ratios for several variables could not be calculated due to zero squares.* | | | | | | | | | | | | |

| **Appendix ii.**  **Bivariate Associations Hospitalizations**  **Table** | **Non-Stratified**  **Bivariate Associations** | | | **Control**  **Bivariate Associations** | | | **Monoclonal Antibody**  **Bivariate Associations** | | | | |
| --- | --- | --- | --- | --- | --- | --- | --- | --- | --- | --- | --- |
|  | **Hospitalization No. (%)**  **(*n*=136)** | **OR (95% CI)** | ***P*** | **Hospitalization No. (%)**  **(*n=*)** | **OR (95% CI)** | ***P*** | **Hospitalization No. (%)**  **(*n*=134)** | **OR (95% CI)** | | ***P*** |  |
| Ethnicity | | 0.0570 | |  | 0.1564 | |  | 0.3004 | | | |
| Latino/Hispanic | 34 (12.6) | -- | | 26 (19.1) | -- | | 8 (6.0) | -- | | |  |
| Other | 0 (0.0) | 1.00 (Referent) | | 0 (0.0) | 1.00 (Referent) | | 0 (0.0) | 1.00 (Referent) | | |  |
| Gender | | 0.0487 | |  | 0.0178 | |  | 0.9306 | | | |
| Male | 23 (8.5) | 2.13 (0.99, 4.56) | | 19 (14.0) | 3.03 (1.18, 7.78) | | 4 (3.0) | 0.94 (0.23, 3.92) | | |  |
| Female | 11 (4.1) | 1.00 (Referent) | | 7 (5.2) | 1.00 (Referent) | | 4 (3.0) | 1.00 (Referent) | | |  |
| Age > 55 | | 0.0127 | |  | 0.0384 | |  | 0.5961 | | | |
| Yes | 32 (11.9) | 5.33 (1.24, 22.93) | | 26 (19.1) | -- | | 6 (4.5) | 1.55 (0.30, 8.03) | | |  |
| No | 2 (0.7) | 1.00 (Referent) | | 0 (0.0) | 1.00 (Referent) | | 2 (1.5) | 1.00 (Referent) | | |  |
| Comorbidities |  | 0.4714 | |  | 0.1528 | |  | 0.4075 | | |  |
| Yes | 29 (10.7) | 1.44 (0.53, 3.93) | | 21 (15.4) | 2.13 (0.74, 6.10) | | 8 (5.6) | -- | | |  |
| No | 5 (1.9) | 1.00 (Referent) | | 5 (3.7) | 1.00 (Referent) | | 0 (0.0) | 1.00 (Referent) | | |  |
| Obesity* |  | 0.9202 | |  | 0.6557 | |  |  | 0.1109 | |  |
| Yes | 7 (2.7) | 0.96 (0.39, 2.34) | | 3 (2.3) | 0.74 (0.20, 2.77) | | 4 (3.1) | 3.31 (0.71, 15.58) | | |  |
| No | 25 (9.7) | 1.00 (Referent) | | 22 (17.2) | 1.00 (Referent) | | 3 (2.3) | 1.00 (Referent) | | |  |
| Diabetes |  | 0.0612 | |  | 0.0810 | |  | 0.1019 | | |  |
| Yes | 19 (7.0) | 1.98 (0.96, 4.10) | | 13 (9.6) | 2.14 (0.90, 5.10) | | 6 (4.5) | 3.63 (0.71, 18.68) | | |  |
| No | 15 (5.6) | 1.00 (Referent) | | 13 (9.6) | 1.00 (Referent) | | 2 (1.5) | 1.00 (Referent) | | |  |
| Hypertension |  | 0.5187 | |  | 0.1490 | |  | 0.4442 | | |  |
| Yes | 22 (8.2) | 1.28 (0.61, 2.71) | | 18 (13.2) | 1.95 (0.78, 4.85) | | 4 (3.0) | 0.58 (0.137, 2.41) | | |  |
| No | 12 (4.4) | 1.00 (Referent) | | 8 (5.9) | 1.00 (Referent) | | 4 (3.0) | 1.00 (Referent) | | |  |
| Coronary Artery Disease |  | 0.8887 | |  | 0.8138 | |  | 0.6106 | | |  |
| Yes | 6 (2.2) | 0.94 (0.37, 2.40) | | 5 (3.7) | 1.14 (0.38, 3.40) | | 1 (0.8) | 0.58 (0.07, 4.91) | | |  |
| No | 28 (10.4) | 1.00 (Referent) | | 21 (15.4) | 1.00 (Referent) | | 7 (5.2) | 1.00 (Referent) | | |  |
| Chronic Kidney Disease |  | 0.1851 | |  | 0.0491 | |  | 0.5277 | | |  |
| Yes | 3 (1.1) | 2.44 (0.63, 9.51) | | 3 (2.2) | 4.65 (0.88, 24.53) | | 0 (0.0) | -- | | |  |
| No | 31 (11.5) | 1.00 (Referent) | | 23 (16.9) | 1.00 (Referent) | | 8 (6.0) | 1.00 (Referent) | | |  |
| Immunodeficiency |  | 0.5164 | |  | 0.0491 | |  | 0.3831 | | |  |
| Yes | 3 (1.1) | 1.54 (0.42, 5.64) | | 3 (2.2) | 4.65 (0.88, 24.53) | | 0 (0.0) | -- | | |  |
| No | 31 (11.5) | 1.00 (Referent) | | 23 (16.9) | 1.00 (Referent) | | 8 (6.0) | 1.00 (Referent) | | |  |
| Cancer |  | 0.0204 | |  | 0.0375 | |  | 0.6089 | | |  |
| Yes | 5 (1.9) | 3.53 (1.14, 10.87) | | 5 (3.7) | 3.50 (1.01, 12.10) | | 0 (0.0) | -- | | |  |
| No | 29 (10.7) | 1.00 (Referent) | | 21 (15.4) | 1.00 (Referent) | | 8 (6.0) | 1.00 (Referent) | | |  |
| Chronic Respiratory Disease |  | 0.8366 | |  | 0.6627 | |  | 0.4542 | | |  |
| Yes | 4 (1.5) | 1.13 (0.37, 3.46) | | 2 (1.5) | 1.44 (0.27, 7.60) | | 2 (1.50) | 1.88 (0.35, 10.00) | | |  |
| No | 30 (11.1) | 1.00 (Referent) | | 24 (17.7) | 1.00 (Referent) | | 6 (4.50) | 1.00 (Referent) | | |  |
| **Obesity has 5 missing observations. P value derived from Chi-square. Odds ratios for several variables could not be calculated due to zero squares.* | | | | | | | | | | | |

| **Appendix iii. Bivariate Associations for Mortality**  **Table** | **Non-Stratified**  **Bivariate Associations** | | | **Control**  **Bivariate Associations** | | | **Monoclonal Antibody**  **Bivariate Associations** | | | |
| --- | --- | --- | --- | --- | --- | --- | --- | --- | --- | --- |
|  | **Mortality No. (%)**  **(*n*=5)** | **OR (95% CI)** | ***P*** | **Mortality No. (%)**  **(*n=*5)** | **OR (95% CI)** | ***P*** | **Mortality No. (%)**  **(*n*=0)** | **OR (95% CI)** | | ***P*** |
| Ethnicity | | 0.4883 | |  | 0.5642 | |  | - | | |
| Latino/Hispanic | 5 (1.9) | -- | | 5 (3.8) | -- | | 0 (0.0) | -- | | |
| Other | 0 (0.0) | 1.00 (Referent) | | 0 (0.0) | 1.00 (Referent) | | 0 (0.0) | -- | | |
| Gender | | 0.1951 | |  | 0.1880 | |  | - | | |
| Male | 4 (1.5) | 3.88 (0.43, 35.18) | | 4 (3.0) | 4.00 (0.44, 36.78) | | 0 (0.0) | -- | | |
| Female | 1 (0.4) | 1.00 (Referent) | | 1 (0.8) | 1.00 (Referent) | | 0 (0.0) | -- | | |
| Age > 55 | | 0.2193 | |  | 0.3993 | |  | - | | |
| Yes | 5 (1.9) | -- | | 5 (3.8) | -- | | 0 (0.0) | -- | | |
| No | 0 (0.0) | 1.00 (Referent) | | 0 (0.0) | 1.00 (Referent) | | 0 (0.0) | -- | | |
| Comorbidities |  | 0.9762 | |  | 0.5702 | |  | - | | |
| Yes | 4 (1.5) | 0.97 (0.11, 8.84) | | 4 (3.0) | 1.89 (0.20, 17.40) | | 0 (0.0) | -- | | |
| No | 1 (0.4) | 1.00 (Referent) | | 1 (0.8) | 1.00 (Referent) | | 0 (0.0) | -- | | |
| Obesity* |  | 0.8949 | |  | 0.7158 | |  |  | - | |
| Yes | 1 (0.4) | 0.86 (0.09, 7.87) | | 1 (0.8) | 1.51 (0.16, 14.38) | | 0 (0.0) | -- | | |
| No | 4 (1.6) | 1.00 (Referent) | | 4 (3.2) | 1.00 (Referent) | | 0 (0.0) | -- | | |
| Diabetes |  | 0.3987 | |  | 0.2565 | |  | - | | |
| Yes | 3 (1.12) | 2.14 (0.35, 13.02) | | 3 (2.3) | 2.77 (0.45, 17.17) | | 0 (0.0) | -- | | |
| No | 2 (0.75) | 1.00 (Referent) | | 2 (1.5) | 1.00 (Referent) | | 0 (0.0) | -- | | |
| Hypertension |  | 0.3469 | |  | 0.2779 | |  | - | | |
| Yes | 4 (1.5) | 2.76 (0.30, 25.05) | | 4 (3.0) | 3.21 (0.35, 29.53) | | 0 (0.0) | -- | | |
| No | 1 (0.4) | 1.00 (Referent) | | 1 (0.8) | 1.00 (Referent) | | 0 (0.0) | -- | | |
| Coronary Artery Disease |  | 0.9234 | |  | 0.8704 | |  | - | | |
| Yes | 1 (0.4) | 1.11 (0.12, 10.20) | | 1 (0.8) | 1.20 (0.13, 11.30) | | 0 (0.0) | -- | | |
| No | 4 (1.5) | 1.00 (Referent) | | 4 (3.0) | 1.00 (Referent) | | 0 (0.0) | -- | | |
| Chronic Kidney Disease |  | 0.0911 | |  | 0.0889 | |  | - | | |
| Yes | 1 (0.4) | 5.70 (0.59, 55.38) | | 1 (0.8) | 6.15 (0.58, 65.57) | | 0 (0.0) | -- | | |
| No | 4 (1.5) | 1.00 (Referent) | | 4 (3.0) | 1.00 (Referent) | | 0 (0.0) | -- | | |
| Immunodeficiency |  | 0.1828 | |  | 0.0516 | |  | - | | |
| Yes | 1 (0.4) | 4.12 (0.43, 39.15) | | 1 (0.8) | 7.75 (0.70, 86.02) | | 0 (0.0) | -- | | |
| No | 4 (1.5) | 1.00 (Referent) | | 4 (3.0) | 1.00 (Referent) | | 0 (0.0) | -- | | |
| Cancer |  | 0.0008 | |  | 0.0086 | |  | 0.6089 | | |
| Yes | 2 (0.8) | 12.77 (1.96, 83.19) | | 2 (1.5) | 8.81 (1.30, 59.72) | | 0 (0.0) | -- | | |
| No | 3 (1.2) | 1.00 (Referent) | | 3 (2.3) | 1.00 (Referent) | | 8 (6.0) | 1.00 (Referent) | | |
| Chronic Respiratory Disease |  | 0.4307 | |  | 0.5642 | |  | 0.4542 | | |
| Yes | 0 (0.0) | -- | | 0 (0.0) | -- | | 2 (1.50) | 1.88 (0.35, 10.00) | | |
| No | 5 (1.9) | 1.00 (Referent) | | 5 (3.8) | 1.00 (Referent) | | 6 (4.50) | 1.00 (Referent) | | |
| **Obesity has 16 missing observations. P value derived from Chi-square. Odds ratios for several variables could not be calculated due to zero squares.* | | | | | | | | | | |
