# Supplementary material for: Bamlanivimab Reduces ED Returns and Hospitalizations and May Reduce COVID-19 Burden on Low-resource Border Hospitals
Source: West J Emerg Med. 2022 Mar 17;23(3):302–11. doi: 10.5811/westjem.2021.10.52668 (PMC9183783; doi:10.5811/westjem.2021.10.52668)
Supplement: Supplementary file 2 [file 52668-APPENDIX_TABLES.docx]

| **Table 1. Comparison of Unexposed Versus Exposed Bamlanivimab Patient Demographics and Characteristics of COVID-19 Emergency Department Patients.** | | | | | |
| --- | --- | --- | --- | --- | --- |
| **Characteristics** | **All**  **N=270^a^** | **No Bamlanivimab (Unexposed)**  **n=136** | **Bamlanivimab (Exposed)**  **n=134** | **p-value** | |
| Age |  |  |  |  | |
| Mean (SD) | 61.7 (13.6) | 63.3 (12.4) | 60.3 (14.7) |  | |
| Median (Min, Max) | 62.0 (19, 93) | 63.0 (20, 93) | 62.0 (19, 91) | 0.0681 | |
|  |  |  |  |  | |
| BMI |  |  |  |  | |
| Mean (SD) | 31.0 (6.6) | 30.2 (4.9) | 31.8 (7.9) |  | |
| Median (Min, Max) | 29.4 (17.1, 61.1) | 29.4 (17.1, 45.6) | 29.4 (21.0, 61.1) | 0.0517 | |
|  |  |  |  |  | |
| Symptom onset  (Days)^b^ |  |  |  |  | |
| Mean (SD) | 4.9 (4.0) | 5.2 (4.5) | 4.6 (3.3) |  | |
| Median (Min, Max) | 4.0 (1.0,28.0) | 4.0 (1.0, 28.0) | 4.0 (1.0,18.0) | 0.2144 | |
|  |  |  |  |  | |
|  | *No. (%)* | *No. (%)* | *No. (%)* |  | |
| Ethnicity |  |  |  |  | |
| Latinx/Hispanic | 247 (91.5) | 128 (94.1) | 119 (88.8) | 0.1180 | |
| Other^d^ | 23 (8.5) | 8 (5.88) | 15 (11.2) |  | |
| Sex |  |  |  |  | |
| Male | 140 (51.9) | 71 (52.2) | 69 (51.5) | 0.9066 | |
| Female | 130 (48.1) | 65 (47.8) | 65 (48.5) |  | |
|  |  |  |  |  | |
| Age > 55 years old | 209 (77.4) | 120 (88.2) | 89 (66.4) | <.0001 | |
|  |  |  |  |  | |
|  | *n=257* | *n=128* | *n=129* |  | |
| BMI > 35^c^ | 58 (22.6) | 19 (14.8) | 30 (30.2) | 0.0032 | |
| Missing | 13 | 8 | 5 |  | |
|  |  |  |  |  | |
| Comorbidities^e^ | 218 (80.7) | 94 (69.1) | 124 (92.5) | <.0001 | |
|  |  |  |  |  | |
| CAD/HLD^f^ | 50 (18.5) | 24 (17.6) | 26 (19.4) | 0.7104 | |
|  |  |  |  |  | |
| HTN | 161 (59.6) | 77 (56.6) | 84 (62.7) | 0.3095 | |
|  |  |  |  |  | |
| DM | 111 (41.1) | 48 (35.3) | 63 (47.0) | 0.0503 | |
|  |  |  |  |  | |
| CKD^g^ | 12 (4.4) | 6 (4.48) | 6 (4.41) | 0.9791 | |
|  |  |  |  |  | |
| Immunocompromised^h^ | 17 (6.3) | 6 (4.41) | 11 (8.21) | 0.1990 | |
|  |  |  |  |  | |
| Cancer | 16 (5.9) | 12 (8.82) | 4 (3.00) | 0.0422 | |
|  |  |  |  |  | |
| CRD^i^ | 29 (10.7) | 8 (5.9) | 21 (15.7) | 0.0094 | |
|  |  |  |  |  | |
| ^a^ Column percentage represented. Total N=270, unexposed patients n=136, exposed patients n=134, unless otherwise specified.  ^b^ Patient reported symptom onset of COVID-19 during evaluation in the ED.  ^c^ Body Mass Index (BMI) missing for 13 patients. Total N=257, unexposed patients n=128, exposed patients n=129.  ^d^ Other ethnicity/race who identified themselves as White, Black, or Asian, or non-Latinx/Hispanic.  ^e^ At least one of the listed comorbidities: diabetes (DM), coronary artery disease/hyperlipidemia (CAD/HLD), hypertension (HTN), chronic kidney disease (CKD), chronic respiratory disease (CRD), immunosuppression, cancer/lymphoma (Cancer).  ^f^ CAD/HLD - History/documented cardiac stents, coronary artery bypass surgery, hyperlipidemia on lipid-lowering agents.  ^g^ History/documented renal failure, peritoneal or hemodialysis.  ^h^ Current immunosuppressive therapy such as steroids, anti-cancer, protein drugs, among others.  ^i^ CRD includes asthma, pulmonary fibrosis, or chronic obstructive pulmonary disease (COPD), among other chronic lung diseases. | | | | |  |

| **Table 2. Clinical Outcomes of COVID-19 Patients No Bamlanivimab Exposure to Bamlanivimab Exposure.** | | | | |
| --- | --- | --- | --- | --- |
| **Outcomes^a^** | **All patients** | **No Bamlanivimab (Unexposed)** | **Bamlanivimab (Exposed)** | **p-value** |
|  | N=270  *No. (%)* | n=136  *No. (%)* | n=134  *No. (%)* |  |
| Return Visit to ED in 14 days | 67 (24.8) | 48 (35.3) | 19 (14.2) | <0.0001 |
| Hospitalization in 14 days | 34 (12.6) | 26 (19.1) | 8 (6.0) | 0.0011 |
|  | *n=266^**^* | *n=132* | *n=134* |  |
| Endotracheal Intubation^b^ | 3 (1.13) | 3 (2.22) | 0 (0) | 0.0862^b^ |
| Missing | 4 | 4 | 0 |  |
|  | *n=267^**^* | *n=133* | *n=134* |  |
| Mortality^b,c^ |  |  |  | 0.0235^b,c^ |
| Survived | 262 (98.2) | 128 (96.2) | 134 (100.0) |  |
| Died | 5 (1.9) | 5 (3.76) | 0 (0.0) |  |
| Missing | 3 | 3 | 0 |  |

^a^ Column percents presented

^b^ Patients were transferred to outside hospitals and therefore records could not be obtained regarding their care. Data is missing for 4 patients requiring endotracheal intubation, total N=266, unexposed patients n=132, exposed patients n=134. Mortality data is missing outcomes of 3 patients, with total N=267, unexposed patients n=132, exposed patients n=134.

^c^ For ventilator and Death outcomes, chi-square analysis is unreliable due to >25% of the data missing in cells.

**Table 3. Full Regression Outcome Models for COVID-19 ED Return Visits in 14 days and Hospitalizations.**

| **Outcome** | **Variable**  **N=257**^a^ | **Estimate** | **Standard Error** | **Likelihood Ratio 95 % CL** | **Wald Chi-Square** | **p-value** |
| --- | --- | --- | --- | --- | --- | --- |
| ED Return Visits within 14 days | Bamlanivimab | -1.11 | 0.317 | -1.75, -0.502 | 12.2 | 0.0005^b^ |
|  | Age ≥ 55 | 0.589 | 0.427 | -0.21, 1.49 | 1.90 | 0.1686 |
|  |  |  |  |  |  |  |
|  | CKD | 1.200 | 0.627 | -0.049, 2.46 | 3.66 | 0.0557^b^ |
|  |  |  |  |  |  |  |
|  | Bamlanivimab | -1.34 | 0.4982 | -2.39, -0.412 | 7.24 | 0.0071^b^ |
|  |  |  |  |  |  |  |
|  | Male | 0.908 | 0.440 | 0.0677, 1.808 | 4.25 | 0.0391^b^ |
|  |  |  |  |  |  |  |
| Hospitalizations | Age ≥ 55 | 1.28 | 0.810 | -0.138, 3.20 | 2.48 | 0.115^c^ |
|  |  |  |  |  |  |  |
|  | Diabetes | 0.686 | 0.464 | -0.205, 1.63 | 2.18 | 0.140^c^ |
|  |  |  |  |  |  |  |
|  | Cancer | 1.13 | 0.743 | -0.370, 2.60 | 2.30 | 0.129^c^ |
|  |  |  |  |  |  |  |
| ^a^ Data missing from 13 patients.  ^b^ Variables were statistically significant at a α =0.1 in bivariate analysis and were included in the full regression model. | | | | | | |
| ^C^ Variables that were significant in the bivariate analysis at a α =0.1, but not found to be statistically significant and were re-entered into the developing models prior to the final reduced model. | | | | | |  |

**Table 4. Final, Reduced Multivariate Regression Outcome Models of ED Visits within 14 days and Subsequent Hospitalizations.**

| **Outcome** | **Variable**  **N=270**^a^ | **Estimate** | **Standard Error** | **Likelihood Ratio**  **95 % CL** | **Wald Chi-Square** | **p-value** |  |
| --- | --- | --- | --- | --- | --- | --- | --- |
| ED Return Visits within 14 days | Bamlanivimab | -1.22 | 0.31 | -0.63, -1.84 | 15.48 | <.0001^b^ |  |
|  | CKD | 1.28 | 0.63 | 0.297, 2.53 | 4.16 | 0.0413^b^ |  |
|  |  |  |  |  |  |  |  |
| Hospitalizations | Bamlanivimab | -1.45 | 0.4339 | - 2.36, -0.639 | 11.14 | 0.0008^b^ |  |
|  |  |  |  |  |  |  |  |
|  | DM | 0.8854 | 0.38 | 1.65, 0.1372 | 5.30 | 0.0213^b^ |  |
|  |  |  |  |  |  |  |  |
| ^a^ No data missing.  ^b^ Variables that reached statistical significance an α =0.05 in the final models.  Those receiving Bamlanivimab on average had 22.83% (mean estimate = 0.7717, CI [0.6482, 0.8611]) less risk of having an ED return visit in 14 days after adjusting for CKD status (p <0.0001 ).  Those receiving Bamlanivimab on average had 19.03% (mean estimate=0.8097, CI [0.6451, 0.9087]) less risk of being hospitalized after adjusting for diabetic status (p =0.0008 ). | | | | | | | |

| **Appendix i. Bivariate Associations ED Return Visits**  **Table** | | **Non-Stratified**  **Bivariate Associations** | | | **Control**  **Bivariate Associations** | | | **Monoclonal Antibody**  **Bivariate Associations** | | | | |
| --- | --- | --- | --- | --- | --- | --- | --- | --- | --- | --- | --- | --- |
|  |  | **Return ED Visit, No. (%)**  **(*n*=136)** | **OR (95% CI)** | ***P*** | **Return ED Visit, No. (%)**  **(*n=*)** | **OR (95% CI)** | ***P*** | **Return ED Visit, No. (%)**  **(*n*=134)** | **OR (95% CI)** | | ***P*** |  |
| Ethnicity |  | 0.0040 | | |  | 0.0313 | |  | 0.0948 | | | |
| Latino/Hispanic | | 67 (24.8) | -- | | 48 (35.3) | -- | | 19 (14.2) | -- | | |  |
| Other | | 0 (0.0) | 1.00 (Referent) | | 0 (0.0) | 1.00 (Referent) | | 0 (0.0) | 1.00 (Referent) | | |  |
| Gender | | | 0.3581 | |  | 0.2907 | |  | 0.9146 | | | |
| Male | | 38 (14.1) | 1.30 (0.74, 2.26) | | 28 (20.6) | 1.47 (0.72, 2.98) | | 10 (7.5) | 1.05 (0.40, 2.79) | | |  |
| Female | | 29 (10.7) | 1.00 (Referent) | | 20 (14.7) | 1.00 (Referent) | | 9 (6.7) | 1.00 (Referent) | | |  |
| Age > 55 | | | 0.0162 | |  | 0.0422 | |  | 0.8418 | | | |
| Yes | | 59 (21.9) | 2.61 (1.17, 5.81) | | 46 (33.8) | 4.35 (0.95, 20.03) | | 13 (9.7) | 1.11 (0.39, 3.15) | | |  |
| No | | 8 (3.0) | 1.00 (Referent) | | 2 (1.5) | 1.00 (Referent) | | 6 (4.5) | 1.00 (Referent) | | |  |
| Comorbidities | |  | 0.9726 | |  | 0.2728 | |  | 0.6937 | | |  |
| Yes | | 54 (20.0) | 0.99 (0.49, 1.99) | | 36 (26.5) | 1.55 (0.71, 3.41) | | 18 (13.4) | 1.53 (0.18, 12.80) | | |  |
| No | | 13 (4.8) | 1.00 (Referent) | | 12 (8.8) | 1.00 (Referent) | | 1 (0.8) | 1.00 (Referent) | | |  |
| Obesity* | |  | 0.6184 | |  | 0.9290 | |  |  | 0.7575 | |  |
| Yes | | 13 (5.1) | 0.84 (0.42, 1.68) | | 7 (5.5) | 1.05 (0.38, 2.88) | | 6 (4.7) | 1.18 (0.41, 3.42) | | |  |
| No | | 51 (19.8) | 1.00 (Referent) | | 39 (30.5) | 1.00 (Referent) | | 12 (9.3) | 1.00 (Referent) | | |  |
| Diabetic | |  | 0.2020 | |  | 0.0575 | |  | 0.5965 | | |  |
| Yes | | 32 (11.9) | 1.44 (0.82, 2.50) | | 22 (16.2) | 2.02 (0.97, 4.18) | | 10 (7.5) | 1.30 (0.49, 3.44) | | |  |
| No | | 35 (13.0) | 1.00 (Referent) | | 26 (19.1) | 1.00 (Referent) | | 9 (6.7) | 1.00 (Referent) | | |  |
| Hypertension | |  | 0.7846 | |  | 0.1663 | |  | 0.0491 | | |  |
| Yes | | 39 (14.4) | 0.93 (0.53, 1.62) | | 31 (22.8) | 1.67 (0.81, 3.44) | | 8 (6.0) | 0.37 (0.14, 1.00) | | |  |
| No | | 28 (10.4) | 1.00 (Referent) | | 17 (12.5) | 1.00 (Referent) | | 11 (8.2) | 1.00 (Referent) | | |  |
| Coronary Artery Disease | |  | 0.3826 | |  | 0.8247 | |  | 0.2909 | | |  |
| Yes | | 10 (3.7) | 0.72 (0.34, 1.52) | | 8 (5.9) | 0.90 (0.35, 2.29) | | 2 (1.5) | 0.45 (0.10, 2.07) | | |  |
| No | | 57 (21.1) | 1.00 (Referent) | | 40 (29.4) | 1.00 (Referent) | | 17 (12.7) | 1.00 (Referent) | | |  |
| Chronic Kidney Disease | |  | 0.0388 | |  | 0.0118 | |  | 0.8582 | | |  |
| Yes | | 6 (2.2) | 3.23 (1.00, 10.38) | | 5 (3.7) | 10.12 (1.15, 89.30) | | 1 (0.8) | 1.22 (0.14, 11.08) | | |  |
| No | | 61 (22.6) | 1.00 (Referent) | | 43 (31.6) | 1.00 (Referent) | | 18 (13.4) | 1.00 (Referent) | | |  |
| Immunodeficiency | |  | 0.8991 | |  | 0.4407 | |  | 0.6136 | | |  |
| Yes | | 4 (1.5) | 0.93 (0.29, 2.95) | | 3 (2.2) | 1.89 (0.37, 9.73) | | 1 (0.8) | 0.58 (0.07, 4.84) | | |  |
| No | | 63 (23.3) | 1.00 (Referent) | | 45 (33.1) | 1.00 (Referent) | | 18 (13.4) | 1.00 (Referent) | | |  |
| Cancer | |  | 0.2258 | |  | 0.6285 | |  | 0.5288 | | |  |
| Yes | | 6 (2.2) | 1.90 (0.66, 5.44) | | 5 (3.7) | 1.35 (0.40, 4.49) | | 1 (0.8) | 2.07 (0.20, 21.05) | | |  |
| No | | 61 (22.6) | 1.00 (Referent) | | 43 (31.6) | 1.00 (Referent) | | 18 (13.4) | 1.00 (Referent) | | |  |
| Chronic Respiratory Disease | |  | 0.7146 | |  | 0.8929 | |  | 0.1683 | | |  |
| Yes | | 8 (3.0) | 1.18 (0.49, 2.79) | | 3 (2.2) | 1.11 (0.25, 4.85) | | 5 (3.7) | 2.21 (0.70, 6.98) | | |  |
| No | | 59 (21.9) | 1.00 (Referent) | | 45 (33.1) | 1.00 (Referent) | | 14 (10.5) | 1.00 (Referent) | | |  |
| **Obesity has 5 missing observations. P value derived from Chi-square. Odds ratios for several variables could not be calculated due to zero squares.* | | | | | | | | | | | | |

| **Appendix ii.**  **Bivariate Associations Hospitalizations**  **Table** | **Non-Stratified**  **Bivariate Associations** | | | **Unexposed**  **No Bamlanivimab**  **Bivariate Associations** | | | **Exposed**  **Bamlanivimab**  **Bivariate Associations** | | | | |
| --- | --- | --- | --- | --- | --- | --- | --- | --- | --- | --- | --- |
|  | **Hospitalization No. (%)**  **(*n*=136)** | **OR (95% CI)** | ***P*** | **Hospitalization No. (%)**  **(*n=*)** | **OR (95% CI)** | ***P*** | **Hospitalization No. (%)**  **(*n*=134)** | **OR (95% CI)** | | ***P*** |  |
| Ethnicity | | 0.0570 | |  | 0.1564 | |  | 0.3004 | | | |
| Latino/Hispanic | 34 (12.6) | -- | | 26 (19.1) | -- | | 8 (6.0) | -- | | |  |
| Other | 0 (0.0) | 1.00 (Referent) | | 0 (0.0) | 1.00 (Referent) | | 0 (0.0) | 1.00 (Referent) | | |  |
| Gender | | 0.0487 | |  | 0.0178 | |  | 0.9306 | | | |
| Male | 23 (8.5) | 2.13 (0.99, 4.56) | | 19 (14.0) | 3.03 (1.18, 7.78) | | 4 (3.0) | 0.94 (0.23, 3.92) | | |  |
| Female | 11 (4.1) | 1.00 (Referent) | | 7 (5.2) | 1.00 (Referent) | | 4 (3.0) | 1.00 (Referent) | | |  |
| Age > 55 | | 0.0127 | |  | 0.0384 | |  | 0.5961 | | | |
| Yes | 32 (11.9) | 5.33 (1.24, 22.93) | | 26 (19.1) | -- | | 6 (4.5) | 1.55 (0.30, 8.03) | | |  |
| No | 2 (0.7) | 1.00 (Referent) | | 0 (0.0) | 1.00 (Referent) | | 2 (1.5) | 1.00 (Referent) | | |  |
| Comorbidities |  | 0.4714 | |  | 0.1528 | |  | 0.4075 | | |  |
| Yes | 29 (10.7) | 1.44 (0.53, 3.93) | | 21 (15.4) | 2.13 (0.74, 6.10) | | 8 (5.6) | -- | | |  |
| No | 5 (1.9) | 1.00 (Referent) | | 5 (3.7) | 1.00 (Referent) | | 0 (0.0) | 1.00 (Referent) | | |  |
| Obesity* |  | 0.9202 | |  | 0.6557 | |  |  | 0.1109 | |  |
| Yes | 7 (2.7) | 0.96 (0.39, 2.34) | | 3 (2.3) | 0.74 (0.20, 2.77) | | 4 (3.1) | 3.31 (0.71, 15.58) | | |  |
| No | 25 (9.7) | 1.00 (Referent) | | 22 (17.2) | 1.00 (Referent) | | 3 (2.3) | 1.00 (Referent) | | |  |
| Diabetes |  | 0.0612 | |  | 0.0810 | |  | 0.1019 | | |  |
| Yes | 19 (7.0) | 1.98 (0.96, 4.10) | | 13 (9.6) | 2.14 (0.90, 5.10) | | 6 (4.5) | 3.63 (0.71, 18.68) | | |  |
| No | 15 (5.6) | 1.00 (Referent) | | 13 (9.6) | 1.00 (Referent) | | 2 (1.5) | 1.00 (Referent) | | |  |
| Hypertension |  | 0.5187 | |  | 0.1490 | |  | 0.4442 | | |  |
| Yes | 22 (8.2) | 1.28 (0.61, 2.71) | | 18 (13.2) | 1.95 (0.78, 4.85) | | 4 (3.0) | 0.58 (0.137, 2.41) | | |  |
| No | 12 (4.4) | 1.00 (Referent) | | 8 (5.9) | 1.00 (Referent) | | 4 (3.0) | 1.00 (Referent) | | |  |
| Coronary Artery Disease |  | 0.8887 | |  | 0.8138 | |  | 0.6106 | | |  |
| Yes | 6 (2.2) | 0.94 (0.37, 2.40) | | 5 (3.7) | 1.14 (0.38, 3.40) | | 1 (0.8) | 0.58 (0.07, 4.91) | | |  |
| No | 28 (10.4) | 1.00 (Referent) | | 21 (15.4) | 1.00 (Referent) | | 7 (5.2) | 1.00 (Referent) | | |  |
| Chronic Kidney Disease |  | 0.1851 | |  | 0.0491 | |  | 0.5277 | | |  |
| Yes | 3 (1.1) | 2.44 (0.63, 9.51) | | 3 (2.2) | 4.65 (0.88, 24.53) | | 0 (0.0) | -- | | |  |
| No | 31 (11.5) | 1.00 (Referent) | | 23 (16.9) | 1.00 (Referent) | | 8 (6.0) | 1.00 (Referent) | | |  |
| Immunodeficiency |  | 0.5164 | |  | 0.0491 | |  | 0.3831 | | |  |
| Yes | 3 (1.1) | 1.54 (0.42, 5.64) | | 3 (2.2) | 4.65 (0.88, 24.53) | | 0 (0.0) | -- | | |  |
| No | 31 (11.5) | 1.00 (Referent) | | 23 (16.9) | 1.00 (Referent) | | 8 (6.0) | 1.00 (Referent) | | |  |
| Cancer |  | 0.0204 | |  | 0.0375 | |  | 0.6089 | | |  |
| Yes | 5 (1.9) | 3.53 (1.14, 10.87) | | 5 (3.7) | 3.50 (1.01, 12.10) | | 0 (0.0) | -- | | |  |
| No | 29 (10.7) | 1.00 (Referent) | | 21 (15.4) | 1.00 (Referent) | | 8 (6.0) | 1.00 (Referent) | | |  |
| Chronic Respiratory Disease |  | 0.8366 | |  | 0.6627 | |  | 0.4542 | | |  |
| Yes | 4 (1.5) | 1.13 (0.37, 3.46) | | 2 (1.5) | 1.44 (0.27, 7.60) | | 2 (1.50) | 1.88 (0.35, 10.00) | | |  |
| No | 30 (11.1) | 1.00 (Referent) | | 24 (17.7) | 1.00 (Referent) | | 6 (4.50) | 1.00 (Referent) | | |  |
| **Obesity has 5 missing observations. P value derived from Chi-square. Odds ratios for several variables could not be calculated due to zero squares.* | | | | | | | | | | | |

| **Appendix iii. Bivariate Associations for Mortality**  **Table** | **Non-Stratified**  **Bivariate Associations** | | | **Control**  **Bivariate Associations** | | | **Monoclonal Antibody**  **Bivariate Associations** | | | |
| --- | --- | --- | --- | --- | --- | --- | --- | --- | --- | --- |
|  | **Mortality No. (%)**  **(*n*=5)** | **OR (95% CI)** | ***P*** | **Mortality No. (%)**  **(*n=*5)** | **OR (95% CI)** | ***P*** | **Mortality No. (%)**  **(*n*=0)** | **OR (95% CI)** | | ***P*** |
| Ethnicity | | 0.4883 | |  | 0.5642 | |  | - | | |
| Latino/Hispanic | 5 (1.9) | -- | | 5 (3.8) | -- | | 0 (0.0) | -- | | |
| Other | 0 (0.0) | 1.00 (Referent) | | 0 (0.0) | 1.00 (Referent) | | 0 (0.0) | -- | | |
| Gender | | 0.1951 | |  | 0.1880 | |  | - | | |
| Male | 4 (1.5) | 3.88 (0.43, 35.18) | | 4 (3.0) | 4.00 (0.44, 36.78) | | 0 (0.0) | -- | | |
| Female | 1 (0.4) | 1.00 (Referent) | | 1 (0.8) | 1.00 (Referent) | | 0 (0.0) | -- | | |
| Age > 55 | | 0.2193 | |  | 0.3993 | |  | - | | |
| Yes | 5 (1.9) | -- | | 5 (3.8) | -- | | 0 (0.0) | -- | | |
| No | 0 (0.0) | 1.00 (Referent) | | 0 (0.0) | 1.00 (Referent) | | 0 (0.0) | -- | | |
| Comorbidities |  | 0.9762 | |  | 0.5702 | |  | - | | |
| Yes | 4 (1.5) | 0.97 (0.11, 8.84) | | 4 (3.0) | 1.89 (0.20, 17.40) | | 0 (0.0) | -- | | |
| No | 1 (0.4) | 1.00 (Referent) | | 1 (0.8) | 1.00 (Referent) | | 0 (0.0) | -- | | |
| Obesity* |  | 0.8949 | |  | 0.7158 | |  |  | - | |
| Yes | 1 (0.4) | 0.86 (0.09, 7.87) | | 1 (0.8) | 1.51 (0.16, 14.38) | | 0 (0.0) | -- | | |
| No | 4 (1.6) | 1.00 (Referent) | | 4 (3.2) | 1.00 (Referent) | | 0 (0.0) | -- | | |
| Diabetes |  | 0.3987 | |  | 0.2565 | |  | - | | |
| Yes | 3 (1.12) | 2.14 (0.35, 13.02) | | 3 (2.3) | 2.77 (0.45, 17.17) | | 0 (0.0) | -- | | |
| No | 2 (0.75) | 1.00 (Referent) | | 2 (1.5) | 1.00 (Referent) | | 0 (0.0) | -- | | |
| Hypertension |  | 0.3469 | |  | 0.2779 | |  | - | | |
| Yes | 4 (1.5) | 2.76 (0.30, 25.05) | | 4 (3.0) | 3.21 (0.35, 29.53) | | 0 (0.0) | -- | | |
| No | 1 (0.4) | 1.00 (Referent) | | 1 (0.8) | 1.00 (Referent) | | 0 (0.0) | -- | | |
| Coronary Artery Disease |  | 0.9234 | |  | 0.8704 | |  | - | | |
| Yes | 1 (0.4) | 1.11 (0.12, 10.20) | | 1 (0.8) | 1.20 (0.13, 11.30) | | 0 (0.0) | -- | | |
| No | 4 (1.5) | 1.00 (Referent) | | 4 (3.0) | 1.00 (Referent) | | 0 (0.0) | -- | | |
| Chronic Kidney Disease |  | 0.0911 | |  | 0.0889 | |  | - | | |
| Yes | 1 (0.4) | 5.70 (0.59, 55.38) | | 1 (0.8) | 6.15 (0.58, 65.57) | | 0 (0.0) | -- | | |
| No | 4 (1.5) | 1.00 (Referent) | | 4 (3.0) | 1.00 (Referent) | | 0 (0.0) | -- | | |
| Immunodeficiency |  | 0.1828 | |  | 0.0516 | |  | - | | |
| Yes | 1 (0.4) | 4.12 (0.43, 39.15) | | 1 (0.8) | 7.75 (0.70, 86.02) | | 0 (0.0) | -- | | |
| No | 4 (1.5) | 1.00 (Referent) | | 4 (3.0) | 1.00 (Referent) | | 0 (0.0) | -- | | |
| Cancer |  | 0.0008 | |  | 0.0086 | |  | 0.6089 | | |
| Yes | 2 (0.8) | 12.77 (1.96, 83.19) | | 2 (1.5) | 8.81 (1.30, 59.72) | | 0 (0.0) | -- | | |
| No | 3 (1.2) | 1.00 (Referent) | | 3 (2.3) | 1.00 (Referent) | | 8 (6.0) | 1.00 (Referent) | | |
| Chronic Respiratory Disease |  | 0.4307 | |  | 0.5642 | |  | 0.4542 | | |
| Yes | 0 (0.0) | -- | | 0 (0.0) | -- | | 2 (1.50) | 1.88 (0.35, 10.00) | | |
| No | 5 (1.9) | 1.00 (Referent) | | 5 (3.8) | 1.00 (Referent) | | 6 (4.50) | 1.00 (Referent) | | |
| **Obesity has 16 missing observations. P value derived from Chi-square. Odds ratios for several variables could not be calculated due to zero squares.* | | | | | | | | | | |

**Appendix iv. Initial Regression Model with All Variables (Factors) for ED Return Visits in 14 days**

| **N=257^a^**  **Variable** |  |  |  |  |  |
| --- | --- | --- | --- | --- | --- |
|  | **Estimate** | **Standard Error** | **Likelihood Ratio 95 % CL** | **Wald Chi-Square** | **p-value** |
| Bamlanivimab^b^ | -1.24 | 0.377 | -1.22, -1.22 | 10.8 | 0.0010^b^ |
| Latinx/Hispanic | 2.75 | 1.46 | 24.3, 24.3 | - | - |
| Male | 0.464 | 0.324 | 0.464, 0.4634 | 2.05 | 0.152 |
| BMI ≥ 35 | 0.241 | 0.414 | 0.241, 0.241 | 0.34 | 0.560 |
| Comorbidities | 0.403 | 0.573 | 0.403, 0.403 | 0.49 | 0.482 |
| Age ≥ 55^b^ | 0.915 | 0.476 | 0.915, 0.915 | 3.69 | 0.0546^b^ |
| CAD/HLD | -0.415 | 0.448 | -0.415, -0.415 | 0.86 | 0.354 |
| DM | 0.369 | 0.367 | 0.369, 0.369 | 1.01 | 0.314 |
| HTN | -0.478 | 0.431 | -0.478, -0.478 | 1.23 | 0.268 |
| CRD | 0.635 | 0.552 | 0.635, 0.635 | 1.33 | 0.249 |
| Immunosuppressed | -0.389 | 0.773 | -0.389, -0.389 | 0.25 | 0.615 |
| Cancer | 0.436 | 0.693 | 0.436, 0.436 | 0.40 | 0.529 |
| CKD^b^ | 1.184 | 0.701 | 1.18, 1.18 | 2.85 | 0.0914^b^ |
| ^a^ Data missing from 13 patients.  ^b^Variables Bamlanivimab exposure, Age ≥ 55, CKD were significant at a α =0.1. All other variables were then removed from the subsequent regression model. | | | | | |

**Appendix v. Intermediary Regression Model with all variables for ED Return Visits in 14 days**

| **N=270^a^**  **Variable** |  |  |  |  |  |
| --- | --- | --- | --- | --- | --- |
|  | **Estimate** | **Standard Error** | **Likelihood Ratio 95 % CL** | **Wald Chi-Square** | **p-value** |
| Bamlanivimab^a^ | -1.11 | 0.317 | -1.75, -0.502 | 12.2 | 0.0005^b^ |
| Age ≥ 55 | 0.589 | 0.427 | -0.21, 1.49 | 1.90 | 0.1686 |
| CKD | 1.200 | 0.627 | -0.049, 2.46 | 3.66 | 0.0557^b^ |
| ^a^ No data missing.  ^b^ Variables Bamlanivimab exposure and were significant at a α =0.1 and will be used in the final, reduced regression model. | | | | | |
|  |  |  |  |  |  |

| **Appendix vi. Initial Regression Model with All Variables (Factors) for Hospitalizations** |
| --- |

| **N=257^a^**  **Variable** |  |  |  |  |  |
| --- | --- | --- | --- | --- | --- |
|  | **Estimate** | **Standard Error** | **Likelihood Ratio 95 % CL** | **Wald Chi-Square** | **p-value** |
| Bamlanivimab^b^ | -1.05 | 0.7523 | -2.53, 0.417 | 1.97 | 0.1600 |
| ED Visit in 14 days^c^ | 28.43 | 0 | 28.43, 28.43 | - | - |
| Latinx/Hispanic^c^ | 0.0023 | 0 | 0.0023, 0.0023 | - | - |
| Male ^b^ | 1.086 | 0.642 | -0.1718, 2.3447 | 2.86 | 0.0906 ^b^ |
| Comorbidities | 1.4081 | 1.1797 | -0.9040, 3.7202 | 1.42 | 0.2326 |
| Age ≥ 55^b^ | 1.4767 | 1.2247 | -0.9236, 3.8771 | 1.45 | 0.22789 |
| CAD/HLD | 0.5294 | 0.8894 | -1.2138, 2.2727 | 0.35 | 0.5517 |
| DM | 0.7348 | 0.6910 | -0.6195, 2.089 | 1.13 | 0.2876 |
| HTN | -1.2474 | 1.0253 | -3.2569, 0.7622 | 1.48 | 0.2238 |
| CRD | -2.840 | 1.1110 | -1.8936, 2.4616 | 0.07 | 0.7982 |
| Immunosuppressed | 1.0459 | 1.8999 | -2.6778, 4.7695 | 0.30 | 0.5820 |
| Cancer | 0.9930 | 1.4404 | -1.8302, 3.8161 | 0.48 | 0.4906 |
| CKD^b^ | -1.1852 | 1.1284 | -3.3969, 1.0264 | 1.10 | 0.2936 |
| ^a^ Data missing from 13 patients.  ^b^Variable Male Sex was significant at a α =0.1.  ^c^Outcomes for ED visit and Latinx/Hispanic variables could not be calculated and had to be removed from the subsequent regression model. | | | | | |
|  |  |  |  |  |  |

**Appendix vii. Intermediary Regression Model with all variables for Hospitalizations.**

| **N=270^a^**  **Variable** |  |  |  |  |  |
| --- | --- | --- | --- | --- | --- |
|  | **Estimate** | **Standard Error** | **Likelihood Ratio 95 % CL** | **Wald Chi-Square** | **p-value** |
| Bamlanivimab^b^ | -1.34 | 0.4982 | -2.388, 0.412 | 7.24 | 0.0071^b^ |
| Male ^b^ | 0.9078 | 0.4401 | 0.0677, 1.808 | 4.25 | 0.0391^b^ |
| Comorbidities | 0.4859 | 0.7869 | -1.045, 2.092 | 0.38 | 0.5370 |
| Age ≥ 55^b^ | 1.2760 | 0.8097 | -0.1382, 3.1957 | 2.48 | 0.1151^b^ |
| CAD/HLD | -0.3522 | 0.5407 | -1.496, 0.654 | 0.42 | 0.5149 |
| DM ^b^ | 0.6858 | 0.4641 | -0.2053, 1.632 | 2.18 | 0.1395^b^ |
| HTN | -0.4090 | 0.5446 | -1.4592, 0.7025 | 0.56 | 0.4527 |
| CRD | 0.2895 | 0.6736 | -1.1453, 1.5549 | 0.18 | 0.6673 |
| Immunosuppressed | -0.1616 | 0.8893 | -2.099, 1.457 | 0.03 | 0.8558 |
| Cancer | 1.1266 | 0.7429 | -0.3698, 2.600 | 2.30 | 0.1294^b^ |
| CKD | 0.3769 | 0.8404 | -1.422, 1.947 | 0.20 | 0.6538 |
| ^a^ Data missing from 13 patients.  ^b^Variables Bamlanivimab exposure, Male, Age, DM, Cancer appears to be significant factors in the modeling, approximate to alpha level 0.1  Removed all variables that were much greater than 0.1. | | | | | |

| **N=270^a^**  **Variable** |  |  |  |  |  |
| --- | --- | --- | --- | --- | --- |
|  | **Estimate** | **Standard Error** | **Likelihood Ratio 95 % CL** | **Wald Chi-Square** | **p-value** |
| Bamlanivimab^b^ | -1.1442 | 0.4484 | -2.081, -0.3040 | 6.51 | 0.0107 ^b^ |
| Male | 0.8149 | 0.4077 | 0.0361, 1.6483 | 3.99 | 0.0457 |
| Age ≥ 55^b^ | 1.1489 | 0.7707 | -0.1620, 3.019 | 2.22 | 0.1360 ^b^ |
| DM | 0.7804 | 0.3946 | 0.0127, 1.5699 | 3.91 | 0.0479 ^b^ |
| Cancer | 1.1101 | 0.6273 | -0.1864, 2.317 | 3.13 | 0.0768 |
| ^a^ No data missing.  ^b^ Variables Bamlanivimab exposure, Male, and DM were significant at a α =0.05 and will be used in the subsequent regression model. | | | | | |

**Appendix viii. Second Intermediary Regression Model with all variables for Hospitalizations.**

**Appendix ix. Fourth Intermediary Regression Model with all variables for Hospitalizations.**

| **N=270^a^**  **Variable** |  |  |  |  |  |
| --- | --- | --- | --- | --- | --- |
|  | **Estimate** | **Standard Error** | **Likelihood Ratio 95 % CL** | **Wald Chi-Square** | **p-value** |
| Bamlanivimab^b^ | -1.4354 | 0.4349 | -2.3486, -0.6241 | 10.89 | 0.0010^b^ |
| Male | 0.7573 | 0.4003 | -0.0080, 1.5748 | 3.58 | 0.0585 |
| DM^b^ | 0.8677 | 0.3870 | 0.1146, 1.6414 | 5.03 | 0.0249^b^ |
| ^a^ No data missing.  ^b^ Variables Bamlanivimab exposure and Diabetes (DM) were significant at a α =0.05 and will be used in the final, reduced regression model. | | | | | |
|  |  |  |  |  |  |
